# Aphid effector Mp10 balances immune suppression and defence activation through EDS1‐dependent modulation of plant DAMP responses

**DOI:** 10.1111/nph.70419

**Published:** 2025-07-30

**Authors:** Matteo Gravino, Sam T. Mugford, Daniela Pontiggia, Joshua Joyce, Claire Drurey, David C. Prince, Felice Cervone, Giulia De Lorenzo, Saskia A. Hogenhout

**Affiliations:** ^1^ Department of Crop Genetics John Innes Centre Norwich Research Park Norwich NR4 7UH UK; ^2^ Department of Biology and Biotechnologies “Charles Darwin” Sapienza University of Rome Piazzale Aldo Moro, 5 00185 Rome Italy; ^3^ Research Centre for Sciences Applied to the Protection of the Environment and Cultural Heritage Sapienza University of Rome Piazzale Aldo Moro, 5 00185 Rome Italy

**Keywords:** *Arabidopsis thaliana*, chemosensory proteins, chemosensory/effector protein 4, damage‐associated molecular pattern‐triggered immunity, effector‐triggered immunity, enhanced disease susceptibility 1, *Myzus persicae*, oligogalacturonides

## Abstract

- Damage‐associated molecular pattern (DAMP)‐triggered immunity (DTI) serves as a crucial first line of defence against aphid attack, yet how aphids manage this response remains unclear.
- By investigating the colonisation of *Arabidopsis thaliana* by the highly polyphagous peach–potato aphid (*Myzus persicae*), we identified cell wall‐derived DAMPs, specifically oligogalacturonides (OGs), as key in inducing DTI against aphids. The OG‐responsive immune components BRASSINOSTEROID INSENSITIVE 1‐ASSOCIATED RECEPTOR KINASE 1/BAK1‐LIKE 1, CPK5/CPK6, GLYCINE‐RICH PROTEIN 3, and EDS1 collectively contribute to DTI limiting aphid colonisation.
- We found that aphid feeding limits OG production in response to wounding. Additionally, the salivary chemosensory protein (CSP) effector Mp10/CSP4, which is known to be delivered into the cytoplasm of plant cells early in aphid attack, inhibits OG‐induced DTI.
- While Mp10 suppresses OG‐induced DTI, it also interacts with EDS1‐mediated defences, enhancing aphid fecundity in the absence of EDS1 while restoring OG responsiveness, revealing its broader role in immune modulation and effector‐driven host adaptation.

## Introduction

Aphids (Hemiptera: Aphididae) are highly specialised sap‐feeding insects and major vectors of economically significant plant viruses. Their ability to colonise a wide range of plants depends on their feeding strategy and molecular interactions with the host. Using their piercing‐sucking stylets, aphids navigate between plant cells, causing minimal tissue damage to reach the phloem sieve elements for sustained feeding (Tjallingii & Esch, 1993). However, this process involves repeated penetration of various plant cells, which can cause mechanical damage and trigger plant defence responses. Plants detect such damage through the release of damage‐associated molecular patterns (DAMPs) that activate DAMP‐triggered immunity (DTI; Tanaka & Heil, 2021; Degli Esposti *et al*., 2025). Yet, aphids appear to suppress these responses to facilitate colonisation. Although aphid feeding has been linked to DTI (Silva‐Sanzana *et al*., 2022), the underlying plant genes remain largely unknown, and the molecular mechanisms aphids use to counteract this defence are poorly understood.

Aphids not only cause direct damage but also play a major role in virus transmission. Viruses can be acquired from various plant tissues and transmitted either by attaching to the inner linings of the stylets and foregut or by circulating throughout the aphid body and being released via saliva (Whitfield *et al*., 2015). Aphids are particularly effective in this role, transmitting nearly 40% of all known vector‐borne plant virus species (Peters *et al*., 2024). Given that most plant viruses rely on vectors for transmission (Ng & Falk, 2006; Hogenhout *et al*., 2008; Ammar *et al*., 2009), understanding plant defences against aphids is essential for developing effective pest and virus management strategies.

Plants have evolved multilayered immune responses to counteract herbivores and pathogens. Pattern recognition receptors (PRRs) on the cell surface detect pathogen‐associated molecular patterns (PAMPs) and DAMPs, triggering PTI/DTI as an initial defence layer (Hou *et al*., 2019). However, successful pathogens and herbivores evade these defences by delivering effectors that suppress PTI/DTI. Intracellular nucleotide‐binding leucine‐rich repeat (NLR) receptors recognise these effectors and trigger a stronger effector‐triggered immunity (ETI), often culminating in localised cell death to restrict further spread (Jones & Dangl, 2006). Recent studies conducted in *Arabidopsis thaliana* have shown that PTI and ETI function in an interconnected manner to provide full resistance, with ENHANCED DISEASE SUSCEPTIBILITY 1 (EDS1) playing a key role in coordinating these immune responses (Ngou *et al*., 2021; Pruitt *et al*., 2021; Tian *et al*., 2021).

Damage perception is a critical component of plant immunity against herbivores (Gatehouse, 2002). Plant cell walls, primarily composed of polysaccharides, such as cellulose, pectin, and hemicelluloses, are continuously monitored for structural integrity (Hamann & Denness, 2011; Molina *et al*., 2024). When cell wall‐derived fragments are released, they act as DAMPs to activate DTI (Aziz *et al*., 2007; Claverie *et al*., 2018; De Lorenzo & Cervone, 2022). Consistent with this, aphid saliva contains enzymes, such as pectin methyl esterase (PME), polygalacturonase (PG), and cellulase (Cherqui & Fred, 2000; Ni *et al*., 2000; Guo *et al*., 2006), which can remodel plant cell walls and potentially promote the release of DAMPs (Molina *et al*., 2024; Pontiggia *et al*., 2024). Moreover, aphid feeding enhances plant PME and pectate lyase activity, leading to modifications in pectin structure (Silva‐Sanzana *et al*., 2019). However, it remains unclear whether aphid‐induced DAMPs are released in sufficient quantities to trigger DTI or how aphids manipulate this process to evade plant defences.

Aphids secrete two types of saliva: ‘sheath’ saliva, which forms a protective barrier around stylets, and ‘watery’ saliva, which is injected into cells during probing (Tjallingii & Esch, 1993). The latter type of saliva contains effectors that modulate plant immunity, including the salivary chemosensory protein (CSP) effector Mp10/CSP4, which is delivered into mesophyll cells during early feeding (Mugford *et al*., 2016). Notably, Mp10 suppresses PTI induced by bacterial flg22 and aphid‐derived elicitors (Bos *et al*., 2010; Drurey *et al*., 2019), potentially by interfering with plant deubiquitinating enzymes, such as ASSOCIATED MOLECULE WITH THE SH3 DOMAIN OF STAM (AMSH), which may lead to destabilisation and mislocalisation of PRRs (Bos *et al*., 2010; Drurey *et al*., 2019; Gravino *et al*., 2024). However, Mp10 also induces chlorosis with cell death‐like features and reduces aphid reproduction (Bos *et al*., 2010; Rodriguez *et al*., 2014; Zhang *et al*., 2023; Rao *et al*., 2024). Mp10 is detected at aphid stylet tips that contain anatomical structures, known as acrostyles, that enable early delivery of effectors and viruses during probing and feeding (Uzest *et al*., 2007; Deshoux *et al*., 2022).

Importantly, aphid performance is negatively affected by treatment with oligogalacturonides (OGs), which are cell wall‐derived DAMPs (Gravino, 2018; Silva‐Sanzana *et al*., 2022). OGs activate immune‐signalling pathways involving BRASSINOSTEROID INSENSITIVE 1‐ASSOCIATED RECEPTOR KINASE 1 (BAK1) and its closest homologue BAK1‐LIKE 1 (BKK1) co‐receptors and calcium‐dependent protein kinases (CDPKs), including CPK5 and CPK6 (Moscatiello *et al*., 2006; Gravino *et al*., 2015, 2017), both of which have been implicated in plant–aphid interactions (Chaudhary *et al*., 2014; Prince *et al*., 2014a; Vincent *et al*., 2017). Moreover, GLYCINE‐RICH PROTEIN 3 (GRP3) also plays a role in OG‐mediated immune signalling (Gramegna *et al*., 2016). Notably, long OGs with a degree of polymerisation (DP) between 10 and 15 are the most active in triggering DTI, whereas short OGs (DP2‐3) exhibit weaker immune activity and can even suppress PTI (Mathieu *et al*., 1991; Vorhölter *et al*., 2012; Gramegna *et al*., 2016; Davidsson *et al*., 2017; Xiao *et al*., 2024). Plants regulate OG signalling through specific OG oxidases that catalyse OG inactivation (Benedetti *et al*., 2018; Pontiggia *et al*., 2020; Salvati *et al*., 2025).

By investigating the colonisation of *A. thaliana* by the highly polyphagous peach–potato aphid (*Myzus persicae*), we show that OG‐induced DTI relies on BAK1/BKK1, CPK5/CPK6, GRP3, and EDS1 to restrict aphid colonisation. Aphids counteract this by limiting OG production in response to wounding and deploying Mp10, which inhibits OG and flg22 sensitivity and PRR stability while simultaneously inducing ETI‐related defences. In EDS1‐deficient plants, Mp10 enhances aphid fecundity, promotes PRR stability, and restores OG and flg22 responsiveness, highlighting a dual role in immune modulation. These findings reveal aphid strategies for evading plant immunity and align with recent discoveries that other aphid effectors actively target EDS1 (Liu *et al*., 2025), further illustrating the complexity of plant–aphid interactions.

## Materials and Methods

### Plant materials

*Arabidopsis thaliana* (Linnaeus; hereafter Arabidopsis) ecotype Columbia‐0 (Col‐0) wild‐type (WT) seeds were purchased from Lehle Seeds. *Nicotiana benthamiana* (Domin) WT seeds were provided by John Innes Centre (JIC) Horticultural Service. Transgenic lines used in this study included the following: Arabidopsis Col‐0 *grp3* and overexpressing *GRP3*‐*RFP* under control of the *Cauliflower mosaic virus* (CaMV) 35S promoter (*GRP3*‐OE #16‐4; Gramegna *et al*., 2016); Arabidopsis Col‐0 *eds1‐2* (Bartsch *et al*., 2006); Arabidopsis Col‐0 *bak1‐5 bkk1‐1* (Schwessinger *et al*., 2011); Arabidopsis Col‐0 *cpk5 cpk6* (Gravino *et al*., 2015) and *cpk5 cpk6 cpk11* (Boudsocq *et al*., 2010); Arabidopsis Col‐0 OG machine (OGM) under control of the β‐oestradiol‐inducible XVE promoter (*XVE:OGM*), and OGM under control of the *PATHOGENESIS‐RELATED GENE 1* (*PR1*) promoter (*pPR1:OGM* #2; Benedetti *et al*., 2015); *N. benthamiana eds1* (Schultink *et al*., 2017); *N. benthamiana adr1 nrg1* (Prautsch *et al*., 2023); *N. benthamiana nrg1‐1* and *nrg1‐2* (Qi *et al*., 2018); and *N. benthamiana* NahG (Wulff *et al*., 2004). The genotype of all Arabidopsis transgenic lines used in this study was confirmed by PCR or, in the case of the *bak1‐5* allele, by sequencing the PCR products (Supporting Information Table S1).

### Generation of dexamethasone‐inducible Mp10 stable transgenic lines

For the generation of stable transgenic Arabidopsis lines expressing *Mp10* (amino acids 23–145) with an N‐terminal Flag‐tag in place of its signal peptide under a dexamethasone (DEX)‐inducible promoter, *Mp10* was amplified from aphid cDNA using a forward primer that encodes a DYKDDDDK (Flag)‐tag and a reverse primer carrying a stop codon (Table S2) and cloned into pBAV150 (Vinatzer *et al*., 2006) using Gateway Technology (Invitrogen, Thermo Fisher Scientific, Altrincham, Cheshire, UK). The recombined plasmid was introduced into *Agrobacterium tumefaciens* strain GV3101‐pMP90RK for subsequent transformation of Arabidopsis Col‐0 WT using the floral dip method (Bechtold *et al*., 1993). Transgenic seeds were selected on phosphinothricin (BASTA). T2 seedlings exhibiting live vs dead segregation ratios of 3 : 1 were taken forward to the T3 stage, and two T3 lines (#7–5 and #9–5) with a 100% survival ratio (considered homozygous) were selected for experiments.

The *eds1‐2* × Mp10 #7–5 and *eds1‐2* × Mp10 #9–5 plants were generated via crossing the above‐mentioned homozygous Mp10 #7–5 and #9–5 lines with the *eds1‐2* mutant in the Col‐0 background (Bartsch *et al*., 2006). Transgenic seeds were selected on BASTA. T3 seedlings with a 100% survival ratio were selected and genotyped by PCR (Chang *et al*., 2019; Table S2) to isolate the T3 lines harbouring the *Mp10* and the *eds1‐2* homozygous alleles.

For experiments with adult plants, 1‐wk‐old Arabidopsis seedlings were transplanted in 8‐cm plastic pots (one seedling per pot) containing 0.4 l compost and grown in a controlled environment room (CER) under a 10 h : 14 h, 22°C : 22°C, light : dark temperature regime and 70% humidity. Plants were used for assays 2 or 3 wk after transplanting.

### Aphids

Stock colonies of *Myzus persicae* (Sulzer) clone O (Mathers *et al*., 2017) were maintained on Arabidopsis plants, ecotype Col‐0 WT, in a CER under a 14 h : 10 h, 24°C : 15°C, light : dark temperature regime and 48% humidity. Age‐synchronised aphids were used for the fecundity assays. To obtain them, *c*. 30 adult female aphids were transferred from the stock colonies to Col‐0 WT 4‐wk‐old plants (5–10 aphids per plant). After 24 h, the adult females were removed from the plants, while the progeny (age‐synchronised nymphs) produced by the females were used for various assays.

### Preparation of standard OGs

OGs enriched in DP10‐15 were prepared from 500 ml solutions containing 2% high molecular weight unmethylated polygalacturonic acid (PGA MW 25000‐50 000; Thermo Fisher Scientific) dissolved in 50 mM sodium acetate pH 5.0 (Sigma‐Aldrich, Gillingham, Dorset, UK), as described previously (Benedetti *et al*., 2017). The PGA solution was transferred into 500 ml Erlenmeyer flasks (100 ml PGA solution per flask) and digested with 0.1 units (U) ml^−1^ of endo‐PGII from *Aspergillus niger* (Sigma‐Aldrich) for 180 min at 30°C with gentle shaking, followed by 10 min incubation in a boiling water bath to inactivate the enzyme. Next, the solutions were cooled down on ice, diluted with cold 50 mM sodium acetate pH 5.0, and supplemented with cold ethanol to a final concentration of 0.5% PGA and 17% ethanol and gently shaken overnight at 4°C. OGs were precipitated from the solutions by centrifugation at 30 000 ***g*** for 30 min. OG pellets, white, were solubilised in 200 ml ultrapure water and centrifuged again at 30 000 ***g*** for 30 min. The supernatant containing OGs was transferred into SpectraPor® dialysis membranes with a molecular weight cut‐off of 1000 Da (Repligen, Boston, MA, USA) and dialysed in ultrapure water and subsequently lyophilised. Approximately 2 g of OG powder was recovered and stored at room temperature. OG stock solutions were freshly prepared in ultrapure water and diluted to 200/250 μg ml^−1^ working solutions for plant immunity assays and high‐performance anion‐exchange chromatography (HPAEC) analyses, respectively.

### Isolation and analysis of OGs from aphid‐infested Arabidopsis leaves

Arabidopsis Col‐0 WT 4‐wk‐old plants were infested with 6‐d‐old aphids (20 or 2 aphids for isolation of OGs after 6 h or after 7/9 d of infestation, respectively) on the first fully expanded leaf from the top and caged inside a clip cage made with transparent plastic tubes and nylon mesh (Prince *et al*., 2014a, 2014b) to minimise the impact of the clip cage on leaf photosynthesis (Kou *et al*., 2022). After 6 hours postinfestation (hpi) or 7/9 day postinfestation (dpi), leaves were excised from plants and aphids were removed using a paintbrush (ESPO, Leicester, UK). Two leaves per treatment were floated in ultrapure water in 6‐well plates (Thermo Fisher Scientific) to remove any trace of insect debris. Controls comprised leaves exposed to empty clip cages and leaves without clip cages (untreated). This experiment was repeated two times.

OGs were isolated with the leaf‐strip method, as described previously (Benedetti *et al*., 2017). Briefly, leaf surfaces were sterilised for 3 min with 4 ml of 1% NaClO and washed four times with 6 ml of sterile ultrapure water. After removing the tip and the petiole, leaves were sliced into 2‐mm‐wide strips using a sterile scalpel blade (Slaughter Ltd, Basildon, Essex, UK) and incubated for 16 h at 30°C with gentle shaking in 2 ml of strong chelating solution (i.e. 50 mM ammonium acetate pH 5.0, 50 mM CDTA, and 50 mM ammonium oxalate; Sigma‐Aldrich) supplemented with 10 mM sodium sulphite (Sigma‐Aldrich) to preserve the OGs and inhibit the activity of OG oxidases during the extraction, or without sodium sulphite, for the eventual detection of oxidised OGs (Benedetti *et al*., 2018). The incubation medium was collected, diluted with 8 ml of 100% ethanol (VWR Chemicals, Lutterworth, Leicestershire, UK), and centrifuged at 15 000 ***g*** for 30 min into an Oak Ridge polypropylene centrifuge tube (Thermo Fisher Scientific). After discarding the supernatant, the pellet was air‐dried inside a laminar flow cabinet, resuspended in 100 μl of ultrapure water, incubated for 20 min at 65°C to eliminate any residual enzymatic activity, and centrifuged at 3000 ***g*** for 3 min to precipitate insoluble material. The clarified supernatant was collected and filtered using nonsterile Costar® Spin‐X® centrifuge tube filters containing cellulose acetate membrane with a pore size of 0.45 μm (Corning, Berlin, Germany).

OGs were analysed by HPAEC using the Ion Chromatography System ICS3000 with pulsed amperometric detection (PAD; Thermo Fischer Scientific), equipped with a Dionex CarboPac PA200 Guard Column (3 × 50 mm; Thermo Fischer Scientific) placed in‐line before a Dionex CarboPac PA200 analytical column (3 × 250 mm; Thermo Fischer Scientific), as described previously (Benedetti *et al*., 2017). Briefly, the columns were equilibrated in a buffer containing 90% eluent A (50 mM potassium hydroxide) and 10% eluent B (50 mM potassium hydroxide and 1 M potassium acetate) at a flow rate of 0.3 ml min^−1^ for 10 min. Then, 10 μl samples containing Arabidopsis‐derived OGs or standard OGs (2.5 μg) were injected and separated by running a linear pump gradient from 90% eluent A and 10% eluent B to 20% eluent A and 80% eluent B over 30 min at the same flow rate. OGs were detected by PAD using a gold working electrode and Waveform A, according to the manufacturer's instructions. The resulting chromatographic peaks, including OG oligomers from DP6 to DP15, were normalised using the compositional data normalisation (CoDA) method, as described previously (Aitchison, 1989; Noonan *et al*., 2018). Briefly, the abundance of OGs was calculated as the log‐ratio of their peak area to the geometric mean (GM) of all peaks in the profile that were shared between different treatments, using the equation:

$${x_{\mathrm{ij}}}^{\text{CoDA}}=\ln\left(\frac{x_{\mathrm{ij}}}{g_{j}}\right)\;\text{where}\;g_{j}=\sqrt[{n}]{x_{1j}\cdot }\;x_{2j}\cdot \ldots x_{\mathrm{nj}}$$

where *x*_*ij*_^CoDA^ is the normalised peak area of the *i* ^th^ peak in the *j* ^th^ profile, *x*_*ij*_ is the *i* ^th^ peak area in the *j* ^th^ profile, and *g*_*j*_ is the GM of all peaks in the *j* ^th^ profile that were shared between different treatments. Because log‐transformation of values lower than 1 generates negative values, transformed data were corrected by adding a constant value, *a* = 4, to obtain all positive values.

### Quantitative reverse transcriptase PCR assays

Arabidopsis Col‐0 WT and DEX‐inducible Mp10 3‐ to 4‐wk‐old plants were sprayed with 1.25 μM DEX or dimethyl sulfoxide (DMSO) as a control. After 3 d, plants were infiltrated with ultrapure water or OGs (200 μg ml^−1^) on the abaxial surface of the first fully expanded leaf from the top. After 2 h, infiltrated leaves were collected, and total RNA was extracted using Tri‐Reagent (Sigma‐Aldrich). Complementary DNA (cDNA) was synthesised from 250 ng of DNase I (RQ1 RNase‐free DNase; Promega, Chilworth, Southampton, UK)‐treated RNA using the reverse transcriptase M‐MLV Kit (Invitrogen, Thermo Fisher Scientific). Reactions of quantitative reverse transcription polymerase chain reaction (qRT‐PCR), consisting of 2.5 ng cDNA, 1 μM of each primer (Table S2), 1X SYBR Green JumpStart Taq ReadyMix (Sigma‐Aldrich), and nuclease‐free water up to 10 μl, were performed in white 96‐well plates (4titude) and detected by a CFX96 Real‐Time System with a C1000 Thermal Cycler (Bio‐Rad, Hercules, CA, USA).

The expression levels of defence‐related genes of interest (GOI), *FLG22‐INDUCED RECEPTOR‐LIKE KINASE 1* (*FRK1*, *At2g19190*), *CYTOCHROME P450*, *FAMILY 81*, *SUBFAMILY F*, *POLYPEPTIDE 2* (*CYP81F2*, *At5g57220*), *PHYTOALEXIN DEFICIENT 3* (*PAD3*, *At3g26830*), and *PHYTOALEXIN DEFICIENT 4* (*PAD4*, *At3g52430*) were normalised to those of housekeeping genes (HKGs) *GLYCERALDEHYDE‐3‐PHOSPHATE DEHYDROGENASE C2* (*GAPDH*, *At1g13440*) and *UBIQUITIN 5* (*UBQ5*, *At3g62250*) as described previously (Pfaffl, 2001; Vandesompele *et al*., 2002; Ferrari *et al*., 2006), with the following modifications. For each gene, the average threshold cycle (aCt) from three technical replicates per biological sample was corrected for the PCR efficiency (E) to obtain Ct using the equation aCT × log(E,2). The GM of Ct values of HKGs was then calculated. The ΔCt between GOIs and HKGs was calculated as Ct_GOI_–GM of Ct_HKGs_. The expression levels for each GOI were calculated as 2^–ΔCT^ to obtain gene expression values relative to *GAPDH* and *UBQ5*.

### Analysing aphid performance on OG‐exposed plants

Arabidopsis WT and mutant/transgenic 4‐wk‐old plants were infiltrated with OGs (200 μg ml^−1^) or ultrapure water, as a control, on the abaxial surface of the first fully expanded leaf from the top. After 72 h, 6‐d‐old age‐synchronised adult aphids were placed on the infiltrated leaf using a moist paintbrush at one aphid in a clip cage per leaf (Prince *et al*., 2014a, 2014b). After 10 d, the numbers of aphids inside each clip cage were counted. For assays with DEX‐inducible Mp10 lines, plants were sprayed with 1.25 μM DEX 3 d before elicitor treatment. Each experiment included 10 plants per sample, unless otherwise stated.

### Aphid whole‐plant fecundity assays

Arabidopsis Col‐0 WT and transgenic 3‐wk‐old plants were seeded with one 1‐d‐old age‐synchronised nymph, individually caged in transparent plastic tubes (10 cm diameter, 30 cm height) capped with white gauze‐covered plastic lids both on top and on the bottom. After *c*. 6 d, the nymphs developed into adults capable of producing their own offspring, which were counted (and removed at each count) on Days 7, 9, and 11 and added up to determine the total number of nymphs produced per adult. Each experiment included eight plants per genotype.

### Chl extraction and quantification in Arabidopsis

Chl extraction and quantification were performed as described previously (Sieber *et al*., 2000). Three‐week‐old Arabidopsis plants, including Col‐0 WT, the *eds1‐2* mutant, and DEX‐inducible Mp10 lines in both Col‐0 and *eds1‐2* backgrounds, were sprayed twice with 1.25 μM DEX at 6‐d intervals. After 12 d, three to five leaves (*c*. 150 mg) were collected from each plant and placed in 15‐ml tubes containing 10 ml of 80% ethanol. Tubes were covered with foil to protect them from light and rotated on a shaker.

Chl content was measured after 24 h of incubation using a NanoDrop 1000 spectrophotometer (Thermo Fisher Scientific) at 647 and 664 nm. The micromolar (μmol) concentration of total Chl was calculated using the following equation and normalised to g of sample fresh weight (Sieber *et al*., 2000):

$$\text{Chlorophyll}\left(\frac{\text{μmol}}{g}\right)=\begin{array}{l} (\left(7.93\cdot \mathrm{Abs}\;\mathrm{at}\;664\;\mathrm{nm}\right)\\ +\;\left(19.53\cdot \mathrm{Abs}\;\mathrm{at}\;647\;\mathrm{nm}\right))/g \end{array}$$

For dark‐induced senescence, the plants were sprayed as described above and then incubated in complete darkness for 5 d before Chl measurements. Whole plants (*c*. 90 mg) were used, incubated in 5‐ml tubes containing 4 ml of 80% ethanol, and processed similarly.

### Detection of hydrogen peroxide by DAB staining in Arabidopsis

Arabidopsis plants were grown and treated as described in the above section. Leaves were detached and placed in 6‐well plates (Thermo Fisher Scientific) containing at least 4 ml of 3,3′‐diaminobenzidine (DAB) staining solution (Daudi & O'Brien, 2012). *In situ* detection of hydrogen peroxide (H_2_O_2_) by DAB staining was performed as described previously (Daudi & O'Brien, 2012). Quantification of DAB staining, expressed as the percentage of total leaf area (excluding the petiole), was conducted using the image processing and the analysis software Fiji (v.2.16.0; Schindelin *et al*., 2012) and the Colour Deconvolution2 plugin (Landini *et al*., 2021), which implements stain unmixing with the Colour Deconvolution plugin (Ruifrok & Johnston, 2001; Methods S1).

### *Agrobacterium tumefaciens*‐mediated transient expression in *N. benthamiana*

*Nicotiana benthamiana* plants were grown in a CER under a 16 h:8 h, 22°C:22°C, light:dark temperature regime and 80% humidity. The constructs containing eGFP‐Mp10 or eGFP alone under control of the CaMV 35S promoter in pB7WGF2 or pB7WG2 plasmids (Karimi *et al*., 2002), respectively, were described previously (Gravino *et al*., 2024). The constructs containing Flag‐Mp10 or Flag alone under control of the CaMV 35S promoter in the pJL‐TRBO plasmid (Lindbo, 2007) were described previously (Gravino *et al*., 2024). The construct containing mCherry alone under control of the CaMV 35S promoter in the pICSL86955OD plasmid (TSL SynBio, Norwich, UK) was described previously (Gravino *et al*., 2024). The construct containing AtFLS2‐3xMyc‐eGFP under control of the *FLS2* promoter in pCAMBIA 2300 was described previously (Robatzek *et al*., 2006). The constructs were introduced into *A. tumefaciens* strain GV3101‐pMP90RK, which was used for agroinfiltration on each side of the abaxial base of two expanded leaves of 4‐ to 5‐wk‐old *N. benthamiana* plants at a final concentration of OD_600_ = 0.3–0.5 in agroinfiltration buffer (10 mM MES pH = 5.5, 10 mM MgCl_2_, and 100 μM acetosyringone) as described previously (Bos *et al*., 2010). To enhance transient gene expression, *A. tumefaciens* carrying the pCB301‐p19 plasmid (Win & Kamoun, 2004) was supplemented to each agroinfiltration mix at OD_600_ = 0.1 (note that this is the OD of just the *A. tumefaciens* clone carrying pCB301‐p19 and not of the entire mixture). This plasmid promotes expression of the p19 protein, a suppressor of post‐transcriptional gene silencing in *N. benthamiana* (Voinnet *et al*., 2003). Leaf/plant assays were conducted 3–14 d postinfiltration (dpi).

### Chlorosis quantification in agroinfiltrated leaf areas of *N. benthamiana*

Chlorosis symptoms in the areas agroinfiltrated with eGFP‐Mp10, but not eGFP alone, became visible at 3 dpi, and images were taken at 7 dpi. Chlorosis was quantified using the Fiji software (v.2.16.0; Schindelin *et al*., 2012) by selecting a region of interest (ROI), including the agroinfiltrated area. The ROI was analysed using a colour histogram to extract the red, green, and blue components. The yellow component, indicative of chlorosis, was calculated using the formula: (0.5 × red) + (0.5 × green).

### Systemic chlorosis quantification and dwarfism phenotyping in *N. benthamiana*

Agrobacteria carrying either Flag‐Mp10 or Flag alone were infiltrated into one side of a single leaf per plant. Systemic chlorosis and dwarfism became visible *c*. 10 dpi, and images were taken at 14 dpi. Systemic chlorosis was observed only in WT plants infiltrated with Flag‐Mp10, primarily in the first two to three leaves from the apex. Chlorosis was quantified in each of these three leaves using the method described above, and the values were averaged to obtain a single measurement per plant. The same procedure was applied to the corresponding leaves of WT plants infiltrated with Flag alone, and to transgenic or mutant lines infiltrated with either construct.

Dwarfism was quantified by measuring plant height using Fiji. For this, after setting the scale to 1 cm, a straight line was drawn from the apex to the soil surface and measured to determine the plant height.

### Protein extraction, immunoblotting, and antibodies

Three days after agroinfiltration, two leaf discs of 7 mm diameter and *c*. 13 mg fresh weight were obtained from the agroinfiltrated area from at least three plants per construct mix (e.g. AtFLS2‐eGFP with Flag alone or Flag‐Mp10) and ground into a fine powder using a TissueLyser LT Bead Mill (Qiagen, Hilden, Germany). The powder was homogenised in 150 μl of protein extraction/loading buffer (10 mM Tris–HCl at pH 7.5, 50 mM dithiothreitol, 4% sodium dodecyl sulphate, 10% glycerol, and 0.05% bromophenol blue), vortexed, boiled for 5 min, and centrifuged at 5000 ***g*** for 3 min. Western blotting was performed as described previously (Gravino *et al*., 2024) using 10 μl of protein extract corresponding to *c*. 0.87 mg of plant tissue. Antibodies and the dilutions used were described previously (Gravino *et al*., 2024), except for anti‐FLS2‐rabbit (PhytoAB, San Jose, CA, USA), which was diluted 1 : 2000.

Western blot bands corresponding to the proteins of interest were quantified using Fiji as described previously (Stael *et al*., 2022) and normalised against the corresponding RuBisCO band in the loading control. Relative changes in protein abundance were calculated as ratios of the normalised densitometric values of protein bands between control and experimental samples.

### ROS burst assays

ROS production was determined using a quantitative luminol/peroxidase‐based method as described previously (Savatin *et al*., 2014a; Gigli‐Bisceglia *et al*., 2015), with some modifications. For ROS burst detection and quantification in Arabidopsis, Col‐0 WT and DEX‐inducible Mp10 3‐ to 4‐wk‐old plants were sprayed with 1.25 μM DEX or DMSO, as a control. After 2 d, at least 24 leaf discs of 4 mm diameter were obtained from at least three plants for each genotype, incubated in white 96‐well plates (Greiner Bio‐One LUMITRAC™, Stonehouse, Gloucestershire, UK) containing 200 μl of ultrapure water per well supplemented with 1.25 μM DEX or DMSO, and covered with aluminium foil. After 1 d, the solutions were replaced with 100 μl of a solution containing 10 μg ml^−1^ horseradish peroxidase (Sigma‐Aldrich) and 17 μg ml^−1^ luminol (Sigma‐Aldrich) supplemented with flg22 peptide (QRLSTGSRINSAKDDAAGLQIA, 100 nM; EZBiolab, Carmel, IN, USA), chitin oligosaccharide (500 μg ml^−1^, Yaizu Suisankagaku Industry, Shizuoka City, Shizuoka, Japan), OGs (200 μg ml^−1^), or water, as a control. OGs and controls were vacuum infiltrated for 2 m before imaging. Luminescence was captured and processed using a Photek camera and provided IFS32 imaging software (Photek, St Leonards on Sea, East Sussex, UK). Luminescence data were acquired every 30 s for at least 30 min, or until no further ROS bursts were detected. Total ROS production was quantified by summing the luminescence values over time as shown in the curves.

For ROS burst detection and quantification in *N. benthamiana*, the same number and size of leaf discs were obtained from the agroinfiltrated area from at least two plants per construct (i.e. eGFP alone or eGFP‐Mp10) and processed as described above.

### Statistical analyses

All statistical analyses were performed in R packages (R Core Team, 2019) or JASP (JASP Team, 2024) using a Student's *t*‐test, one‐way or two‐way ANOVA with *post hoc* Tukey Honestly Significant Difference (HSD) test. Experiments consisted of at least three biological replicates and were repeated at least two times on different days to generate data from at least two independent experiments, which were displayed as plots using R packages (R Core Team, 2019).

## Results

### OG‐triggered immunity reduces *M. persicae* fecundity on Arabidopsis via BAK1/BKK1‐, CPK5/6‐, and GRP3‐mediated defence pathways

**Fig. 1:**
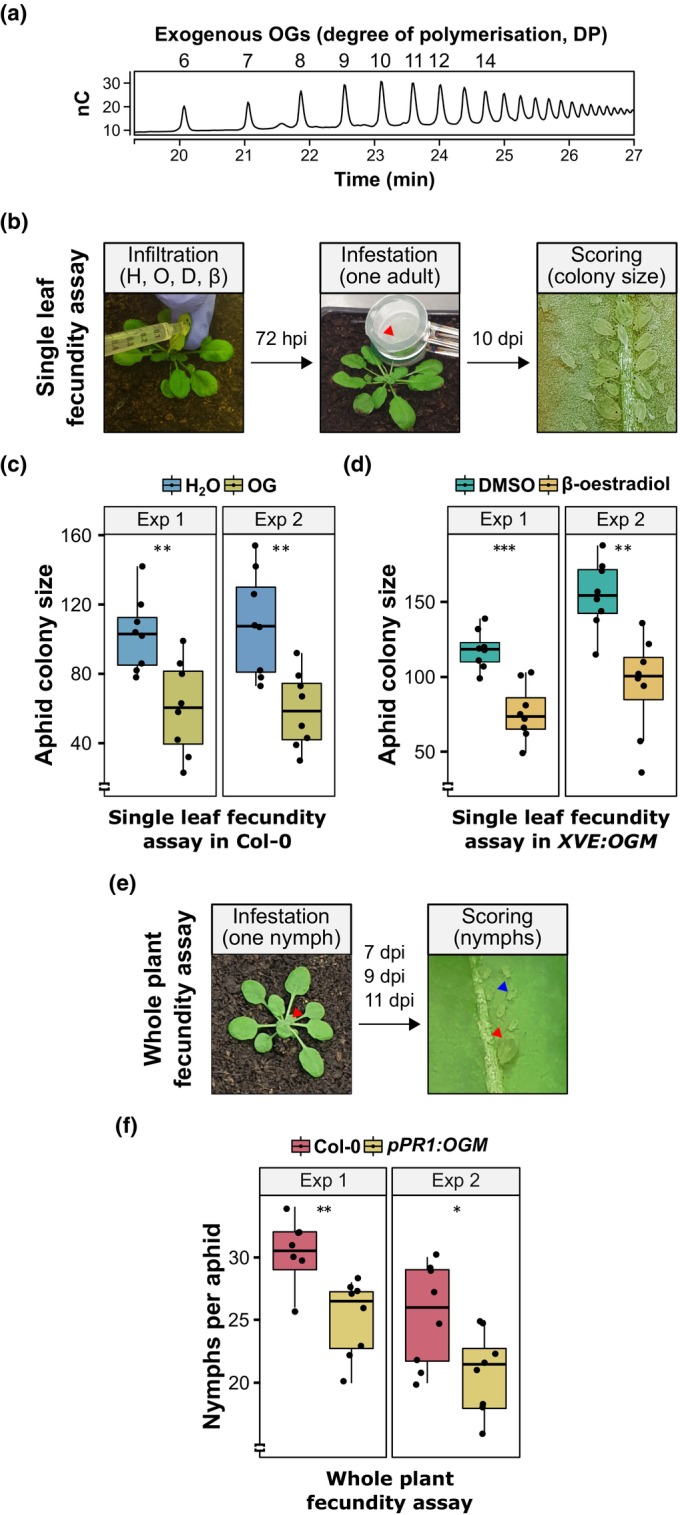
Exogenous and endogenous oligogalacturonides (OGs) increase *Arabidopsis thaliana* resistance against *Myzus persicae*. (a) Profile of exogenous OGs (2.5 μg) enriched in degree of polymerisation (DP) 10–15 used in this work, analysed by high‐performance anion‐exchange chromatography (HPAEC) with pulsed amperometric detector (PAD). The x‐axis shows the retention time in min. The y‐axis shows the detector response in nanocoulombs (nC). (b) Experimental design of aphid single‐leaf fecundity assay. One leaf from 4‐wk‐old *A. thaliana* Columbia‐0 (Col‐0) wild‐type (WT) or *XVE:OGM* transgenic plants was infiltrated with H_2_O (H) and 200 μg ml^−1^ OGs (O), or dimethyl sulfoxide (DMSO) (d) and 5 μM β‐oestradiol (β), respectively (left panel). Seventy‐two hours postinfiltration (hpi), one 6‐d‐old asexually reproducing *M. persicae* adult female was caged on the infiltrated leaf (red arrowhead, middle panel). Ten days postinfestation (dpi), the number of individual aphids (adults + nymphs) within the cage was counted to obtain the aphid colony size (right panel). (c) Aphid single‐leaf fecundity assay in Col‐0 WT plants pretreated with OGs or H_2_O as a control. The y‐axis shows the aphid colony size. (d) Aphid single‐leaf fecundity assay in *XVE:OGM* transgenic plants pretreated with β‐oestradiol or DMSO as a control. The y‐axis shows the aphid colony size. (e) Experimental design of aphid whole‐plant fecundity assay. Arabidopsis Col‐0 WT and *pPR1:OGM* transgenic 3‐wk‐old plants were infested with one 1‐d‐old *M. persicae* nymph (red arrowhead, left panel). The progeny (blue arrowhead, right panel) of this aphid (red arrowhead, right panel) was counted at 7, 9, and 11 dpi and removed from the plant and added up to obtain the number of nymphs produced per individual aphid. (f) Aphid whole‐plant fecundity assay in Col‐0 WT and *pPR1:OGM* transgenic plants. The y‐axis shows the number of nymphs per aphid. In (c, d, f), boxplots show the median, the 25^th^ and 75^th^ percentiles, the most extreme data points (whiskers' extensions), and the observations as black‐filled circles. Data points above the top whisker or below the bottom whisker are outliers. Two independent experiments (exp) are shown. *n* = 8 in each experiment. Asterisks indicate significant differences between samples as determined by Student's *t*‐test (*, *P* < 0.05; **, *P* < 0.01; ***, *P* < 0.001).

We examined whether plant immunity triggered by OG DAMPs plays a role in plant defence against aphids. We found that the fecundity of clonally (asexually) reproducing *M. persicae* aphids was reduced on Arabidopsis Col‐0 WT leaves infiltrated with elicitor‐active exogenous OGs enriched in DP 10–15 (Benedetti *et al*., 2017; Fig. 1a–c) compared with leaves infiltrated with water (Fig. 1b,c). We also measured aphid fecundity on transgenic Arabidopsis plants expressing a polygalacturonase‐inhibiting protein‐PG chimaera, referred to as the ‘OG machine’ (OGM), under control of the *XVE* β‐oestradiol‐inducible promoter (*XVE:OGM*; Benedetti *et al*., 2015; Fig. S1a). Upon treatment with 50 μM β‐oestradiol dissolved in DMSO, these plants accumulated elicitor‐active OGs *in vivo* (Benedetti *et al*., 2015), leading to the activation of DTI (Benedetti *et al*., 2015; Fig. S2a). Before performing aphid fecundity assays on *XVE:OGM* plants, we tested whether β‐oestradiol itself influences aphid reproduction. Treatment with 50 μM β‐oestradiol significantly reduced aphid population growth compared with DMSO in Col‐0 WT plants (Fig. S2b). However, lower concentrations (0.5 and 5 μM) had no significant effect on aphid fecundity in Col‐0 WT plants (Fig. S2b). Based on these results, we selected 5 μM β‐oestradiol for further experiments. At this concentration, β‐oestradiol was sufficient to induce *OGM* transcript accumulation, activate DTI, and significantly reduce aphid fecundity in the *XVE:OGM* plants compared with DMSO‐treated controls (Figs 1b,d, S2a). It is well known that the expression of *PR1* is induced in Arabidopsis ecotypes, such as Col‐0 infested with *M. persicae* (Moran & Thompson, 2001; Kusnierczyk *et al*., 2007; Kettles *et al*., 2013). Hence, we used the *PR1* promoter to drive the OG production by the chimaeric *OGM* gene (Benedetti *et al*., 2015). We found that aphid fecundity was reduced on *pPR1:OGM* plants (Fig. S1a), compared with Col‐0 WT plants (Fig. 1e,f). These results indicate that *M. persicae* fecundity is negatively affected by OG‐induced DTI, in agreement with previous findings (Gravino, 2018; Silva‐Sanzana *et al*., 2022).

**Fig. 2:**
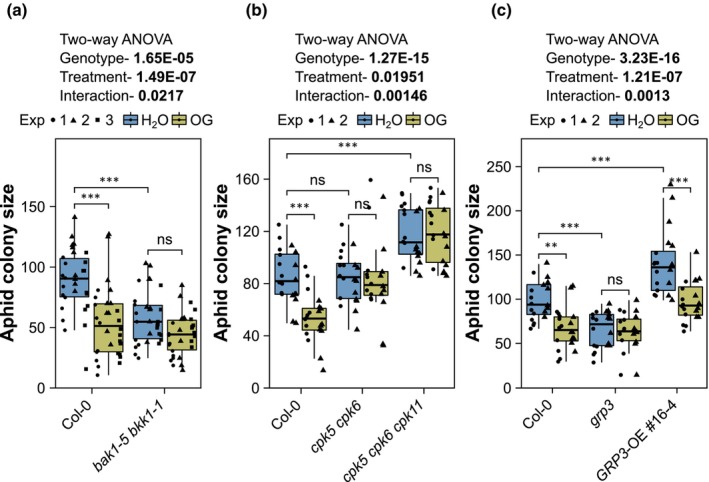
Oligogalacturonide (OG)‐induced protection against *Myzus persicae* in *Arabidopsis thaliana* requires BRASSINOSTEROID INSENSITIVE 1‐ASSOCIATED RECEPTOR KINASE 1 (*BAK1*) and *BAK1‐like* (*BKK1*), *calcium‐dependent protein kinases* (CDPKs) CPK5 and *CPK6*, and *glycine‐rich protein 3* (*GRP3*). (a–c) Aphid single‐leaf fecundity assays. Four‐wk‐old plants of *A. thaliana* Columbia‐0 (Col‐0) wild‐type (WT) and *bak1‐5 bkk1‐1*, *cpk5 cpk6*, *cpk5 cpk6 cpk11*, and *grp3* mutants, and in GRP3‐OE transgenic plants, as indicated, were infiltrated with H_2_O or 200 μg ml^−1^ OG. Seventy‐two h postinfiltration, one 6‐d‐old asexually reproducing *M. persicae* adult female was caged on the infiltrated leaf. After 10 d, the number of individual aphids (adults + nymphs) within the cage was counted to obtain the aphid colony size. The y‐axis shows the aphid colony size. The x‐axis shows the plant genotype. Boxplots show the median, the 25^th^ and 75^th^ percentiles, the most extreme data points (whiskers' extensions), and the observations as black‐filled circles (experiment (exp) 1), triangles (exp 2), or squares (exp 3). Data points above the top whisker or below the bottom whisker are outliers. *n* = 10 in each experiment. Asterisks indicate significant differences between samples as determined by two‐way ANOVA with interaction (genotype:treatment) and *post hoc* Tukey HSD test (**, *P* < 0.01; ***, *P* < 0.001; ns, not significant).

DTI induction by OGs is dependent on key immune‐related elements, including BAK1 and BKK1, CPK5 and CPK6, and GRP3 (Gravino *et al*., 2015; Gramegna *et al*., 2016; Gravino *et al*., 2017; Table S3). Here, we assessed whether these immune‐signalling components affect the OG‐induced reduction of aphid fecundity on Arabidopsis using well‐characterised mutant lines. BAK1 and BKK1 play a redundant and equal contribution in OG immune signalling, as OG‐induced DTI is affected only in the *bak1‐5 bkk1‐1* double mutant, but not in either *bak1‐5* or *bkk1‐1* single mutants (Gravino *et al*., 2017). We therefore assessed aphid performance on the *bak1‐5 bkk1‐1* double mutant (Schwessinger *et al*., 2011; Figs S1b). Unlike Col‐0 WT plants, aphid fecundity was not reduced on OG‐pretreated compared with water‐pretreated *bak1‐5 bkk1‐1* double mutant plants (Fig. 2a). This finding suggests that BAK1 and BKK1 are essential for the OG‐induced reduction of aphid fecundity on Arabidopsis, consistent with their established role as positive regulators of plant immunity (Roux *et al*., 2011). Nonetheless, *bak1‐5 bkk1‐1* mutant plants were overall more resistant to aphids than Col‐0 WT plants (Fig. 2a). This is remarkable, given that this mutant was found to be more susceptible to bacterial and oomycete pathogens (Roux *et al*., 2011).

CDPKs have a role in OG‐induced DTI downstream of BAK1 (Gravino *et al*., 2015). Because CPK5, CPK6, and CPK11 have redundant functions, as OG‐induced DTI is affected only in the *cpk5 cpk6* double and *cpk5 cpk6 cpk11* triple mutants, but not in the respective single mutants (Gravino *et al*., 2015), we assessed aphid performance on the *cpk* double and triple mutants (Fig. S1c). The OG pretreatment did not reduce aphid fecundity on *cpk5 cpk6* double and *cpk5 cpk6 cpk11* triple mutants (Fig. 2b), whereas aphid fecundity was reduced on the OG‐pretreated Col‐0 WT plants (Fig. 2b). Moreover, the aphids produced more progeny on the *cpk5 cpk6 cpk11* triple mutant vs Col‐0 WT (Fig. 2b). These data indicate that the three CPKs play redundant roles in mediating basal and OG‐induced resistance of Arabidopsis to *M. persicae*.

GRP3 is a negative regulator of OG‐induced DTI (Gramegna *et al*., 2016). Consequently, an Arabidopsis *grp3 null* mutant is more resistant to *Botrytis cinerea* (Gramegna *et al*., 2016). In our experiments, aphids produced less progeny on the *grp3 null* mutant (Fig. S1d) compared with Col‐0 WT plants (Fig. 2c), indicating that the *grp3 null* mutant is also more resistant to *M. persicae*. Moreover, compared with Col‐0 WT plants, the aphids produced more progeny on an Arabidopsis line that overexpressed *GRP3* as a fusion to *RFP* under control of the *35S* promoter (*GRP3*‐OE #16–4; Fig. S1c; Gramegna *et al*., 2016; Fig. 2c). The OG pretreatment did not affect aphid fecundity on the *grp3 null* mutant but reduced aphid fecundity on the *GRP3*‐OE #16–4 and Col‐0 WT plants, as compared to water‐pretreated plants (Fig. 2c). These data suggest that GPR3 positively affects the OG‐induced reduction of aphid fecundity on Arabidopsis and acts as a susceptibility factor in the basal resistance of Arabidopsis to *M. persicae*.

Altogether, these results show that BAK1/BKK1, CPK5/CPK6, and GRP3 play an essential role in the OG‐induced DTI that reduces the ability of *M. persicae* to colonise Arabidopsis plants.

### Aphid feeding minimises *in vivo* accumulation of long OGs upon wounding in Arabidopsis

**Fig. 3:**
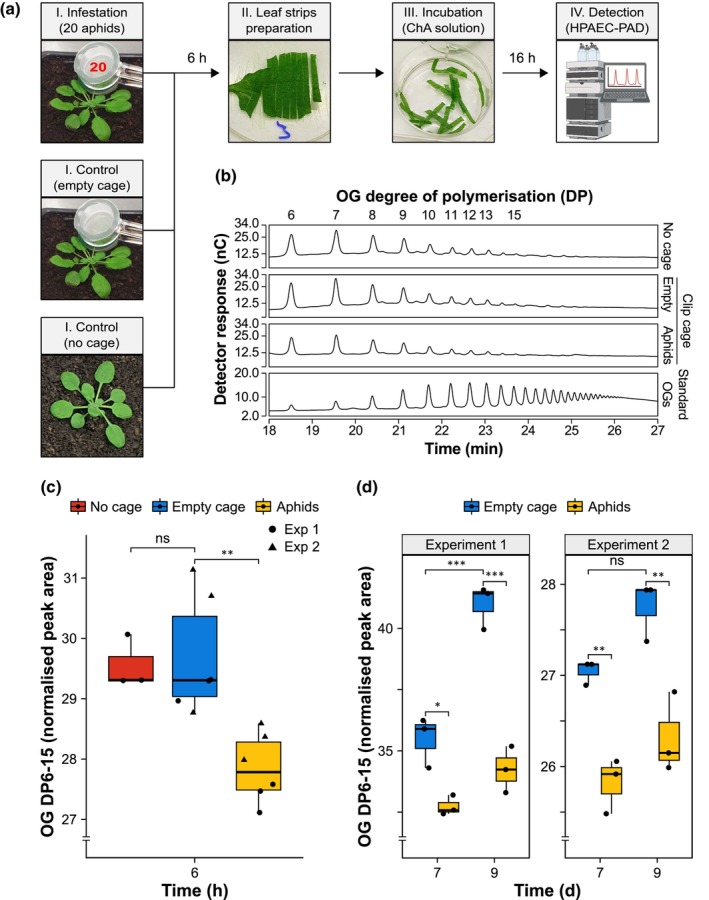
*In vivo* accumulation of long oligogalacturonides (OGs) is suppressed during *Myzus persicae* colonisation of *Arabidopsis thaliana*. (a) Experimental design. Leaves from 4‐wk‐old Arabidopsis Columbia‐0 (Col‐0) wild‐type (WT) plants were caged with 20 6‐d‐old asexually reproducing *M. persicae* adult females, without aphids (empty cage), or not caged, as a control (panels I). After 6 h, leaves were excised and, after removing all aphids, sliced into strips (panel II). Leaf strips were incubated with strong chelating agents supplemented with sodium sulphite (ChA solution) for 16 h (panel III). The leaf diffusates in incubation medium were analysed by high‐performance anion‐exchange chromatography (HPAEC) with a pulsed amperometric detector (PAD; panel IV); this figure was created with BioRender.com (https://BioRender.com). (b) Chromatographic analyses show the profile of OGs across samples. The numbers on top of the graph indicate the degree of polymerisation (DP) of different OG oligomers and refer to the corresponding peaks below. The x‐axis shows the retention time in min. The y‐axis shows the detector response in nanocoulombs (nC). For each sample, the result of one representative replicate out of three performed is shown. The bottom panel shows the profile of 2.5 μg of standard OGs enriched in DP10‐15. (c, d) Total content of OGs (DP6‐15) in control samples and aphid‐infested leaves for (c) 6 h or (d) 7 and 9 d. The x‐axis shows the time of infestation in h or d. The y‐axis shows the peak area (nC min) of total OGs from DP6 to DP15, normalised using the compositional data normalisation (CoDA) method. Boxplots show the median, the 25^th^ and 75^th^ percentiles, the most extreme data points (whiskers' extensions), and the observations as black‐filled circles. Two independent experiments are shown. *n* = 3 in each experiment. Asterisks indicate significant differences between samples as determined by one‐way ANOVA with *post hoc* Tukey HSD test (*, *P* < 0.05; **, *P* < 0.01; ***, *P* < 0.001, ns, not significant).

Given the critical role of OGs in plant defences against *M. persicae*, it remains unclear how aphid feeding affects their accumulation. To address this, we quantified the effect of aphid feeding on the capacity of the plant to produce OGs. We measured OG abundance in leaf diffusates leaking from leaf strips; this method of wounding induces the production and release of OGs (Savatin *et al*., 2014b). Extracts were prepared from Arabidopsis Col‐0 WT leaves previously infested for 6 h with aphids, confined to individual leaves using clip cages, compared with empty clip cages or untreated controls (Fig. 3a). This experimental setup was chosen based on evidence that pectin changes occur in Arabidopsis within 6 h of *M. persicae* infestation (Silva‐Sanzana *et al*., 2019) and that aphids typically settle on a new Arabidopsis plant, that is, reach the phloem feeding phase following transfer, by 3–6 h (Giolai, 2019).

HPAEC‐PAD analysis of leaf diffusates identified peaks in all samples that eluted with retention times comparable to those of an OG solution enriched in long OGs with DP10‐15 (Benedetti *et al*., 2017; Fig. 3b), which most effectively induce plant defences (Mathieu *et al*., 1991; Ferrari *et al*., 2013). However, quantification of the area under the peaks and data normalisation showed that total OG levels with DP6‐15 were lower in diffusates collected from aphid‐infested leaves compared with clip cage controls, with no differences observed between diffusates collected from leaves treated with or without empty clip cages (Figs 3c, S3a).

To account for the possible impact of prolonged aphid feeding on OG release, OG abundance was also assessed after 7 and 9 d of aphid infestation, corresponding to the time points of aphid fecundity assays (Fig. 1b,f). In these experiments, Arabidopsis Col‐0 WT leaves were infested with clip cages containing two adult aphids or exposed to empty clip cages as a control (Fig. S3b). However, despite an average of 30–38 aphids produced per female by these time points (Fig. S3c), total OG levels with DP6‐15 were consistently lower in diffusates collected from aphid‐infested leaves compared with controls after both 7 and 9 d (Figs 3d, S3d,e). In one experiment, we identified OGs with DP3, which exhibit a weaker immune activity (Davidsson *et al*., 2017), and observed that their levels were also reduced in diffusates collected from aphid‐infested leaves compared with controls after both 7 and 9 d (Fig. S3e). Notably, we detected higher levels of OGs in control leaves sampled at 9 d compared with those sampled at 7 d (Fig. 3d), as observed previously in plants at different developmental stages and potentially due to cell wall remodelling associated with plant growth and development (Pontiggia *et al*., 2015, 2020). Shifted peaks potentially corresponding to oxidised OGs that are known not to activate DTI (Benedetti *et al*., 2018) were not detectable in any of our experiments (Fig. S4a–d).

In all our experiments, some of the peaks that were eluting earlier compared with those known to contain OGs with DP10‐15 were increased in aphid‐infested leaves (Fig. S3a,e). To investigate whether the increased peaks (e.g. peak #5, Fig. S3a) represented other cell wall‐derived elicitors, we attempted their identification using mass spectrometry. However, we were unable to detect any compound corresponding to these peaks, likely due to decomposition or poor ionisation during analysis, indicating that they do not contain OGs. Hence, we do not know what compounds underlie these peaks.

In summary, defence‐inducing OGs are released in Arabidopsis aphid‐infested leaves upon wounding but in lower amounts than in noninfested leaves.

### The aphid effector Mp10 suppresses the OG‐triggered ROS burst, defence gene induction, and resistance against *M. persicae*

**Fig. 4:**
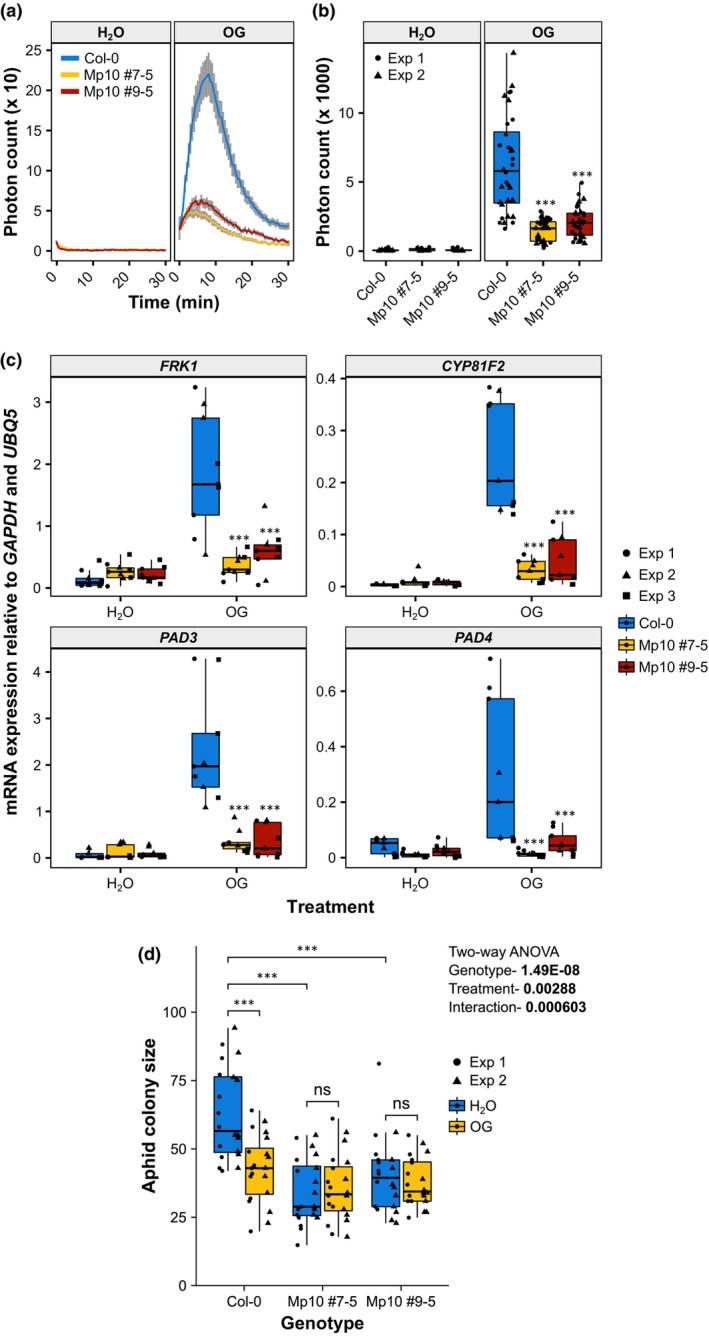
Aphid effector Mp10 suppresses the oligogalacturonide (OG)‐triggered immunity in *Arabidopsis thaliana*. In (a–d), 3‐ to 4‐wk‐old plants of *A. thaliana* Columbia‐0 (Col‐0) wild‐type (WT) and dexamethasone (DEX)‐inducible Mp10 transgenic lines (#7–5 and #9–5) were sprayed with 1.25 μM DEX 3 d before treatment with 200 μg ml^−1^ OG and H_2_O as a control. (a) Reactive oxygen species (ROS) production measured in leaf discs over a period of 30 min after elicitor treatment. Solid line, mean ± SE. *n* = 16 and 32 leaf discs treated with H_2_O and OGs, respectively, from two independent experiments. The x‐axis shows the time in min after elicitor treatment. The y‐axis shows the photon count. (b) Total ROS production over a period of 30 min after elicitor treatment. *n* = 8 and 16 leaf discs treated with H_2_O and OGs, respectively, in each experiment. The y‐axis shows the total photon count. The x‐axis shows the plant genotype. (c) Transcript levels of *FLG22‐INDUCED RECEPTOR‐LIKE KINASE 1* (*FRK1*), *CYTOCHROME P450*, *FAMILY 81*, *SUBFAMILY F*, *POLYPEPTIDE 2* (*CYP81F2*), *PHYTOALEXIN DEFICIENT 3* (*PAD3*), and *PHYTOALEXIN DEFICIENT 4* (*PAD4*) relative to *GLYCERALDEHYDE 3‐PHOSPHATE DEHYDROGENASE* (*GAPDH*) and *UBIQUITIN 5* (*UBQ5*; y‐axis) measured 2 h after elicitor infiltration. *n* = 3 in each experiment. (d) Aphid single‐leaf fecundity assay. Seventy‐two h postelicitor infiltration, one 6‐d‐old asexually reproducing *M. persicae* adult female was caged on the infiltrated leaf. *n* = 10 in each experiment. After 10 d, the number of individual aphids (adults + nymphs) within the cage was counted to obtain the aphid colony size. The y‐axis shows the aphid colony size. The x‐axis shows the plant genotype. In (b–d), boxplots show the median, the 25^th^ and 75^th^ percentiles, the most extreme data points (whiskers' extensions), and the observations as black‐filled circles in experiment (exp) 1, triangles in exp 2, or squares in exp 3. Data points above the top whisker or below the bottom whisker are outliers. Asterisks indicate significant differences between samples as determined by one‐way ANOVA or two‐way ANOVA with interaction (genotype : treatment) and *post hoc* Tukey HSD test, as indicated (***, *P* < 0.001; ns, not significant).

The aphid effector Mp10 is delivered into the cytoplasm of plant mesophyll cells during the early probing phase of aphid feeding (Mugford *et al*., 2016) and has been shown to suppress plant PTI responses (Bos *et al*., 2010; Drurey *et al*., 2019; Gravino *et al*., 2024). Here, we investigated whether Mp10 also suppresses DTI responses. In Arabidopsis Col‐0 WT leaves, OGs induced typical DTI responses, including a peak of ROS within 10 min following elicitation returning to near‐basal levels after 30 min (Fig. 4a), that contributed to the total ROS productions (Fig. 4b), in agreement with previous data (Gravino *et al*., 2015, 2017). Moreover, treatment with OGs induced the expression of the defence‐related genes *FRK1*, *CYP81F2*, *PAD3*, and *PAD4* (Fig. 4c), as reported previously (Denoux *et al*., 2008; Gravino *et al*., 2015, 2017). To investigate whether Mp10 suppresses OG‐induced ROS and defence genes, we generated stable transgenic plants that express Mp10 under the control of a DEX‐inducible promoter in the Arabidopsis Col‐0 background. Two independent homozygous lines (#7–5 and #9–5) that showed *Mp10* expression upon treatment with DEX (solubilised in DMSO) and not with DMSO alone were selected (Fig. S5a,b). However, despite detectable *Mp10* transcripts, we were unable to detect the corresponding proteins in DEX‐pretreated plants using our specific Mp10 antibodies. Nonetheless, we proceeded to phenotype the lines using DMSO as a control and assessed their capacity to activate or suppress plant immunity. ROS kinetics over time and total ROS productions, as well as the expression of the defence genes *FRK1*, *CYP81F2*, *PAD3*, and *PAD4* in response to OGs, were reduced in the DEX‐inducible Mp10 lines pretreated with 1.25 μM DEX compared with Col‐0 plants pretreated with 1.25 μM DEX (Fig. 4a–c), indicating that Mp10 suppresses the ROS burst and defence genes induced by OGs. As a control, OG‐triggered DTI remained unaffected in the DEX‐inducible Mp10 lines in the absence of DEX pretreatment (Fig. S6a–c).

Because PTI/DTI depends on an expanding repertoire of PRRs, many of which, but not all, converge on a small set of coreceptor kinases, such as BAK1 (Yasuda *et al*., 2017), and because BAK1 plays a key role in aphid resistance (Prince *et al*., 2014a; Vincent *et al*., 2017), we investigated whether Mp10 selectively suppresses immune signalling pathways that require BAK1. To test this, we assessed whether Mp10 inhibits ROS burst responses triggered by bacterial flg22 and fungal chitin, which activate BAK1‐dependent and BAK1‐independent immune signalling, respectively (Shan *et al*., 2008). In Mp10‐expressing lines pretreated with 1.25 μM DEX, ROS bursts induced by flg22 were significantly reduced compared with Col‐0 plants similarly pretreated with DEX (Fig. S7a). By contrast, chitin‐induced ROS bursts were unaffected in Mp10 lines (Fig. S7b), aligning with previous findings (Bos *et al*., 2010). These results confirm that Mp10 specifically suppresses BAK1‐dependent immune‐signalling pathways while leaving BAK1‐independent pathways intact.

To test whether the suppression of the OG‐triggered DTI by Mp10 favours aphid colonisation, we measured aphid fecundity in Arabidopsis Col‐0 WT and Mp10‐expressing plants pretreated with 1.25 μM DEX before treatment with or without OGs. Both Mp10‐expressing lines (#7–5 and #9–5) were overall more resistant to aphids than Col‐0 WT plants (Fig. 4d), in agreement with previous data (Bos *et al*., 2010). Moreover, in contrast to Col‐0 WT plants, aphid fecundity was not further reduced on OG‐treated compared with water‐treated Mp10 lines (Fig. 4d), suggesting that Mp10 suppresses the OG‐induced reduction of aphid fecundity on Arabidopsis. The absence of an additive effect indicates that OGs and Mp10 are most likely operating through the same pathway.

### Mp10 induces immune responses that are differentially dependent on EDS1, ADR1, NRG1, and salicylic acid

**Fig. 5:**
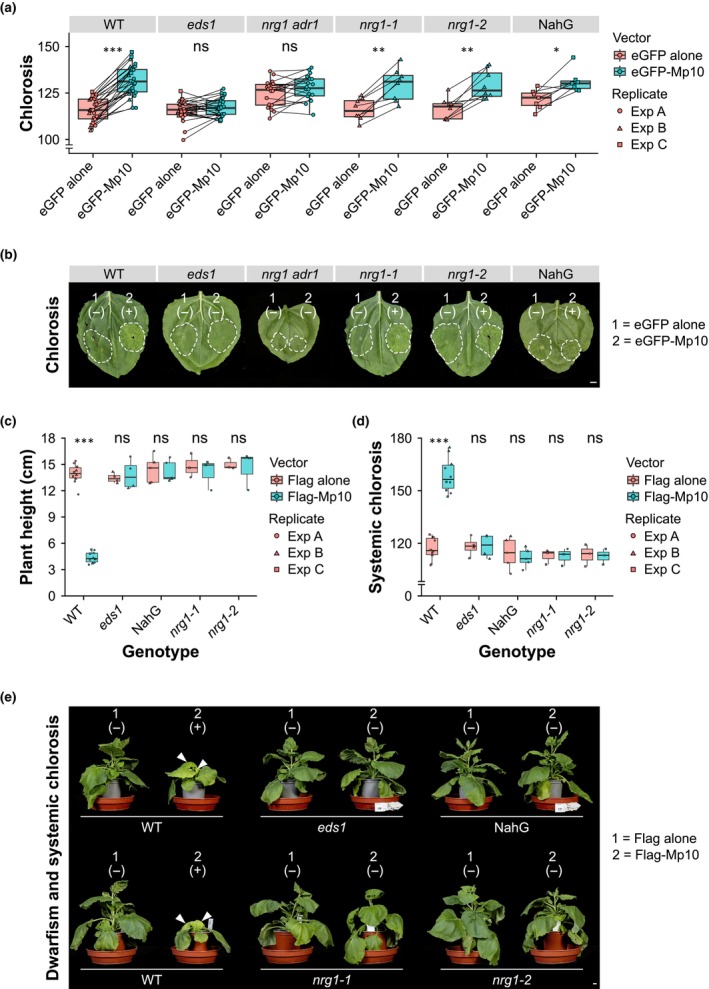
Aphid effector Mp10 promotes effector‐triggered immunity (ETI)‐like responses in *Nicotiana benthamiana* that are differentially dependent on ENHANCED DISEASE SUSCEPTIBILITY 1 (EDS1), ACTIVATED DISEASE RESISTANCE 1 (ADR1), N REQUIREMENT GENE 1 (NRG1), and salicylic acid. (a) Chlorosis measurements in *N. benthamiana* leaves from wild‐type (WT) and the indicated mutants agroinfiltrated with either eGFP alone or eGFP‐Mp10 (x‐axis). Chlorosis, defined as the yellowing of normally green leaf tissue, is quantified on the y‐axis as the average of the red and green colour components in the agroinfiltrated areas. Data points from opposite sides of the same leaf, agroinfiltrated with either eGFP alone or eGFP‐Mp10, are connected by lines in paired boxplots. (b) Representative pictures of leaf patches showing presence (+) or absence (−) of presence (+) or absence (−) of chlorosis after agroinfiltration with either eGFP alone (1) or eGFP‐Mp10 (2) on each side of one leaf per plant (WT and mutants). Pictures were taken 7 d postinfiltration (dpi). (c, d) Plant height and systemic chlorosis measurements in WT and mutant plants agroinfiltrated with either Flag alone or Flag‐Mp10. The x‐axes show the plant genotype. The y‐axes show the plant height (c), defined as the distance between the apex (tip) and the base (at the soil surface) of the stem measured in centimetres (cm), and systemic chlorosis (d), quantified as the average of the red and green colour components in the systemic leaves. (e) Representative plants of WT and indicated mutants showing systemic chlorosis and dwarfism (+) or not (−) after agroinfiltration with either Flag alone (1) or Flag‐Mp10 (2) on one leaf side. White arrowheads indicate systemic leaves with the most severe symptoms, observed only in WT plants agroinfiltrated with Flag‐Mp10. Pictures were taken at 14 dpi. (a, c, d) Boxplots show the median, the 25^th^ and 75^th^ percentiles, the most extreme data points (whiskers' extensions), and the observations as circles in experiment (exp) A, triangles in exp B, or squares in exp C. Data points above the top whisker or below the bottom whisker are outliers. *n* ≥ 3. For each genotype, asterisks indicate significant differences between treatments (vectors) as determined by two‐way ANOVA with interaction (genotype : treatment) and *post hoc* Tukey HSD test, as indicated (***, *P* < 0.001; **, *P* < 0.01; *, *P* < 0.05; ns, not significant). (b, e) Bars, 1 cm.

Since Mp10 induces immunity in a salicylic acid glucosyltransferase 1‐, recognition of CSPs (RCSP)‐, and EDS1‐dependent manner (Bos *et al*., 2010; Rao *et al*., 2024), we further investigated whether ACTIVATED DISEASE RESISTANCE 1 (ADR1), N REQUIREMENT GENE 1 (NRG1), and salicylic acid (SA) are involved in this pathway. Transient production of Mp10 via agroinfiltration in *N. benthamiana* WT leaves induced localised chlorosis in the infiltrated areas observed as early as 3 dpi (Fig. 5a,b; Bos *et al*., 2010; Drurey, 2015). This was followed by systemic chlorosis in uninfiltrated leaves and an overall dwarf phenotype at *c*. 14 dpi (Figs 5c–e, S8a,b; Bos *et al*., 2010; Drurey, 2015; Rao *et al*., 2024). The local chlorosis was observed in the *N. benthamiana nrg1‐1* and *nrg1‐2* mutants and partially in NahG plants (Fig. 5a,b) – NahG encodes a bacterial salicylate hydroxylase that destroys SA (Gaffney *et al*., 1993). However, it was absent in *eds1* and *adr1 nrg1* mutants (Fig. 5a,b). By contrast, systemic chlorosis and dwarfism were not observed in *N. benthamiana nrg1‐1*, *nrg1‐2*, *eds1* mutants, or NahG plants (Figs 5c–e, S8a,b).

Similarly, in Arabidopsis Mp10 transgenic lines, we observed chlorosis/senescence, dwarfism, and intracellular H_2_O_2_ accumulation, as detected by DAB staining. These symptoms were more pronounced in the Mp10 line #7–5 compared with line #9–5, which expresses lower levels of *Mp10* (Fig. S5a,b) and only exhibited symptoms under stress conditions (e.g. continuous darkness; Fig. S9a–j). To investigate whether EDS1 has a role in Mp10‐mediated immune activation also in Arabidopsis, we generated *eds1‐2* × Mp10 lines by genetic crosses (Fig. S1e). Notably, these phenotypes were absent in Arabidopsis *eds1‐2* × Mp10 lines, as well as in WT and *eds1‐2* mutant plants (Fig. S9a–j).

These findings indicate that Mp10 triggers ETI‐like responses that are dependent on EDS1, ADR1, NRG1, and SA.

### Mp10 modulates EDS1‐associated processes as part of the DTI response while also activating ETI

**Fig. 6:**
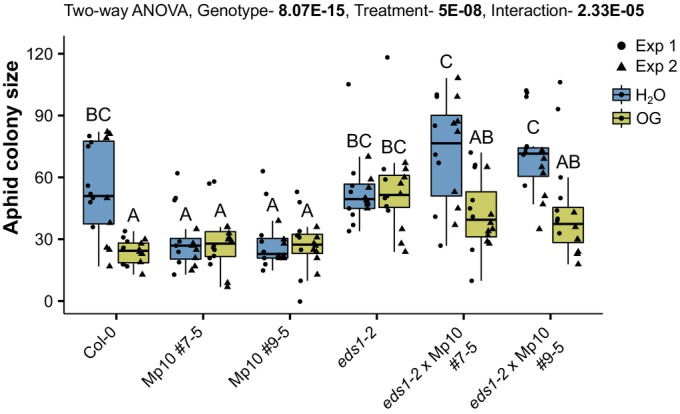
Aphid effector Mp10 promotes aphid resistance and suppresses the oligogalacturonide (OG)‐induced protection against *Myzus persicae* in an ENHANCED DISEASE SUSCEPTIBILITY 1 (*EDS1*)‐dependent manner in *Arabidopsis thaliana*. Aphid fecundity assay. Three‐ to four‐week‐old plants of *A. thaliana* Columbia‐0 (Col‐0) wild‐type (WT) and dexamethasone (DEX)‐inducible Mp10 transgenic lines (#7–5 and #9–5), *eds1‐2* mutant, *eds1‐2* × Mp10 #7–5, and *eds1‐2* × Mp10 #9–5 crosses, as indicated, were sprayed with 1.25 μM DEX and, 3 d later, infiltrated with H_2_O and 200 μg ml^−1^ OG. Seventy‐two h postinfiltration, one 6‐d‐old asexually reproducing *M. persicae* adult female was caged on the infiltrated leaf. After 10 d, the number of individual aphids (adults + nymphs) within the cage was counted to obtain the aphid colony size. The y‐axis shows the aphid colony size. The x‐axis shows the plant genotype. Boxplots show the median, the 25^th^ and 75^th^ percentiles, the most extreme data points (whiskers' extensions), and the observations as black‐filled circles in experiment (exp) 1 or triangles in exp 2. Data points above the top whisker or below the bottom whisker are outliers. *n* = 8 samples in each experiment. Different letters indicate significant differences between samples as determined by two‐way ANOVA with interaction (genotype : treatment) and *post hoc* Tukey HSD test. See Supporting Information Table S4 for *P*‐values.

Since Mp10 triggers immunity in Arabidopsis in an EDS1‐dependent manner, we further examined its impact on aphid resistance in the absence of EDS1. Aphids produced significantly more progeny on the *eds1‐2* × Mp10 lines compared with Mp10 lines (Fig. 6), consistent with our finding that EDS1 is required for Mp10‐induced ETI (Figs 5, S9). Aphids produced more progeny on *eds1‐2* × Mp10 lines compared with WT plants (Fig. 6), although the differences were not statistically significant when the two *eds1‐2* × Mp10 lines were analysed individually. However, when the presence of Mp10 was considered as a combined factor, aphid performance on the *eds1‐2* × Mp10 lines was significantly higher compared with WT and to *eds1‐2* Col‐0 plants (*P* = 0.0208 and *P* = 0.013, respectively, one‐way ANOVA with Tukey HSD).

We next evaluated the role of EDS1 and of Mp10 in the absence of EDS1 in the OG‐induced immunity to aphids. Aphids showed reduced fecundity on OG‐treated WT plants compared with water‐treated WT plants, but their performance remained similar on water‐ and OG‐treated Arabidopsis *eds1‐2* plants (Fig. 6), indicating that OG‐induced DTI depends on EDS1. This finding is consistent with previous reports demonstrating a role for EDS1 in signalling downstream of certain PRRs in Arabidopsis (Pruitt *et al*., 2021). Here, we extend this role to OG‐induced signalling, suggesting that EDS1 is also required for the defence response triggered by OGs. Aphid performances on the water‐ and OG‐treated Mp10 lines were similar and comparable to those of OG‐treated WT plants (Fig. 6), in agreement with previous data (Fig. 4d). Surprisingly, however, aphids did less well on OG‐treated than on water‐treated *eds1‐2* × Mp10 lines (Fig. 6), indicating that the OG‐mediated resistance response to aphids was present in the *eds1‐2* × Mp10 lines, unlike the *eds1‐2* lines.

Overall, these data indicate that Mp10 promotes aphid fecundity in the absence of EDS1 and that EDS1 is required for OG‐induced immunity against aphids. Yet paradoxically, Mp10 compensates for the loss of EDS1, restoring plant responsiveness to OGs. Therefore, Mp10 operates within the EDS1 pathway, modulating not only EDS1‐associated ETI but also DTI responses.

### Mp10 fails to destabilise PRRs and suppress PTI in the absence of EDS1

**Fig. 7:**
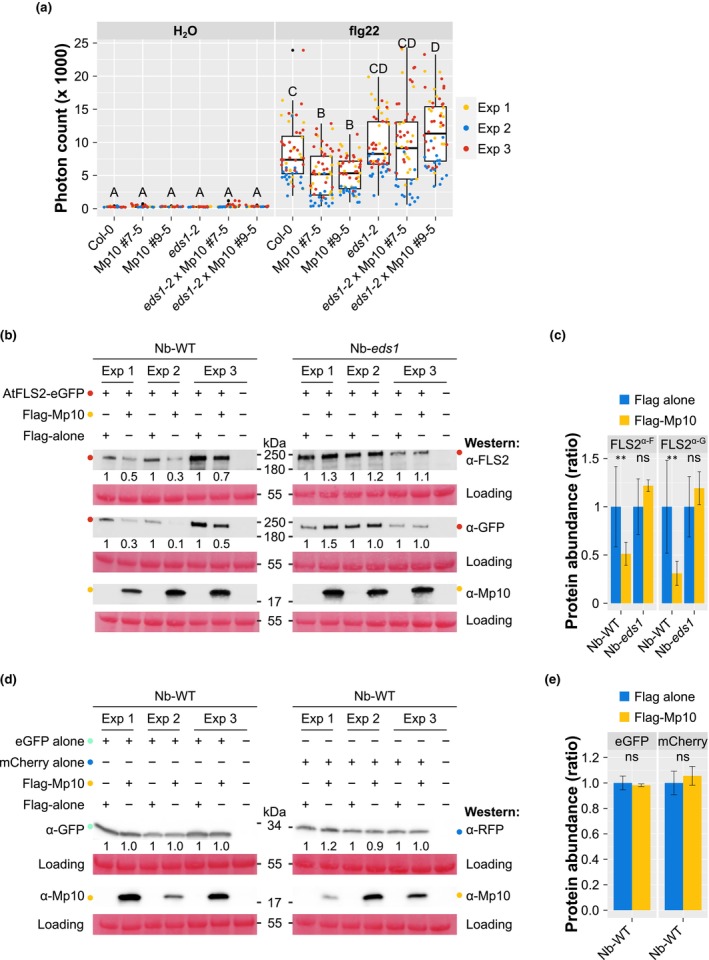
ENHANCED DISEASE SUSCEPTIBILITY 1 (EDS1) is required for Mp10‐mediated suppression of the reactive oxygen species (ROS) burst induced by bacterial flg22 in *Arabidopsis thaliana* and the destabilisation of FLS2. (a) Total ROS production over 8 h following treatment with 100 nM flg22 or H₂O (control) in 4‐wk‐old plants of *A. thaliana* Col‐0 wild‐type (WT), *eds1‐2* mutant, and dexamethasone (DEX)‐inducible Mp10 transgenic lines (Mp10 #7–5 and #9–5) and crosses (*eds1‐2* × Mp10 #7–5 and #9–5) pretreated with 1.25 μM DEX. Y‐axis: total photon count; X‐axis: plant genotype. Boxplots display the median, 25^th^ and 75^th^ percentiles, whiskers indicating extreme data points, and observations as circles coloured in yellow for experiment (exp) 1, blue for exp 2, or red for exp 3. Data points above the top whisker or below the bottom whisker are outliers. *n* = 8 and *n* ≥ 16 leaf discs per H_2_O and flg22 treatment, respectively, per experiment. Different letters indicate significant differences between samples as determined by two‐way ANOVA with interaction (genotype : treatment) and Tukey HSD (*P* < 0.001). (b, d) Western blot analyses of *Nicotiana benthamiana* WT or *eds1* mutant leaf discs co‐agroinfiltrated with eGFP alone, mCherry alone, or *A. thaliana* FLS2‐eGFP and either Flag alone or Flag‐Mp10, as indicated. Proteins were detected using antibodies against FLS2, eGFP, RFP, and Mp10, as indicated. MW markers are shown between blots, with expected bands marked by coloured dots (red, AtFLS2‐eGFP; light green, eGFP alone; yellow, Flag‐Mp10; blue, mCherry alone). Within each exp, relative changes in AtFLS2‐eGFP, eGFP alone, or mCherry alone stability are shown below the blots as ratios of the normalised densitometric values of protein bands between control (in the presence of Flag alone) and experimental (in the presence of Flag‐Mp10) samples. Loading control (Ponceau S staining) was used for normalisation. (c, e) Quantitative analysis of AtFLS2‐eGFP, eGFP alone, or mCherry alone abundance as detected with antibodies against FLS2 (α‐F), GFP (α‐G), or RFP in the presence of Flag alone or Flag‐Mp10 in *N. benthamiana* WT or *eds1* mutant, as indicated. Y‐axis, protein abundance as a ratio between control and experimental samples; X‐axis, plant genotype. Results are shown as the mean of the three replicates shown in (b, d) ± SE. Asterisks indicate significant differences between control and experimental samples as determined by ANOVA with *post hoc* Tukey HSD test (**, *P* < 0.01; ns, not significant).

Our finding that Mp10 may act in the EDS1 node instigated further investigations of the involvement of EDS1 in Mp10 immune‐suppressing activities. Since it is unclear which PRRs perceive OGs (Herold *et al*., 2024), we used the well‐characterised flg22/FLS2 system, as it was previously found that flg22 treatment induces the expression of immune genes effective against aphids (Kettles *et al*., 2013; Prince *et al*., 2014a) and Mp10 affects the stability of the flg22 receptor FLS2 (Gravino *et al*., 2024). We found that the flg22‐triggered ROS burst was reduced in the Arabidopsis Mp10 lines compared with Col‐0 plants (Fig. 7a), confirming our previous data (Fig. S7a). However, this suppression was not observed in the *eds1‐2* × Mp10 lines compared with the *eds1‐2* mutant (Fig. 7a), suggesting that EDS1 is required for Mp10‐mediated suppression of the flg22‐induced ROS burst. Consistent with previous findings in Arabidopsis (Pruitt *et al*., 2021), ROS responses to flg22 were similar between Col‐0 and *eds1‐2* plants (Fig. 7a).

Additionally, transient production of eGFP‐Mp10 suppressed the flg22‐induced ROS burst in WT *N. benthamiana* plants, compared with an eGFP alone control, as observed previously (Bos *et al*., 2010; Gravino *et al*., 2024), whereas this Mp10‐mediated suppression activity was not observed in *N. benthamiana eds1* plants (Fig. S10). Importantly, EDS1 is not known to impact flg22‐elicited ROS production in *N. benthamiana* (Fig. S10; Zönnchen *et al*., 2022). These findings prompted us to further examine whether Mp10's effects on FLS2 stability are also dependent on EDS1, using the *N. benthamiana* transient expression system, which allows efficient protein production and detection and comparison between control and experimental samples in the same leaf. Flag‐Mp10 destabilised Arabidopsis FLS2‐eGFP in WT *N. benthamiana*, consistent with previous findings (Gravino *et al*., 2024), but not in *N. benthamiana eds1* plants (Fig. 7b,c). We examined the accumulation of nonplant proteins to control for the possibility that Mp10 broadly affects *A. tumefaciens*‐mediated protein expression, as previously reported (Rodriguez *et al*., 2014). In contrast to the previous reports (Rodriguez *et al*., 2014), we did not observe Mp10 generally affecting *A. tumefaciens*‐mediated transient protein expression, as the levels of eGFP alone and mCherry alone were not reduced in the presence of Flag‐Mp10 relative to the Flag‐alone control (Fig. 7d,e), suggesting that Mp10 does not affect *A. tumefaciens*‐mediated transient protein expression.

Together, these data indicate that EDS1 is required for the Mp10‐mediated ROS suppression and PRR destabilisation activities.

## Discussion

**Fig. 8:**
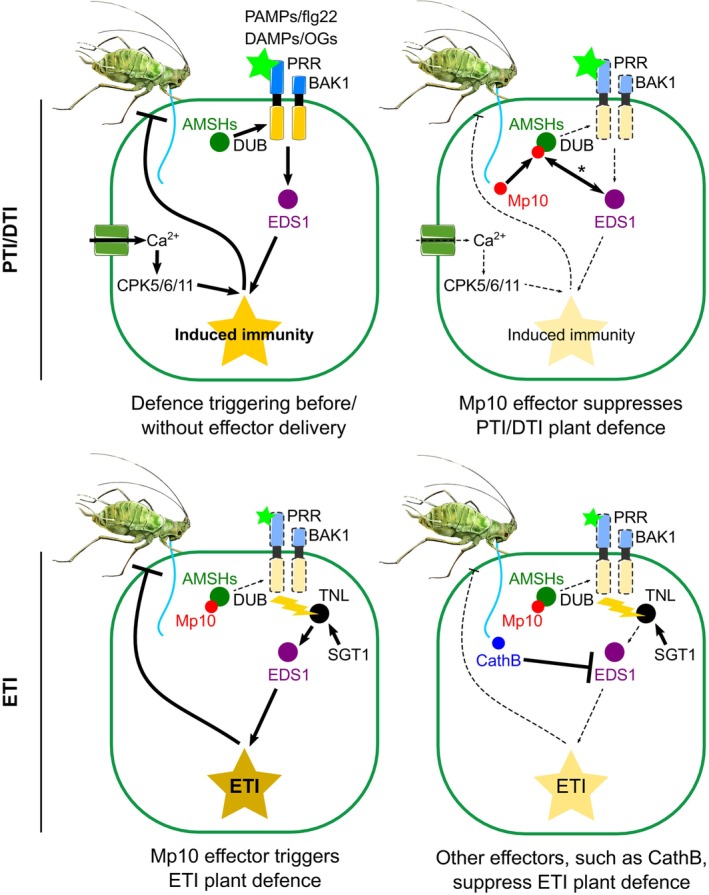
Model: Activation and suppression of defences in plant–aphid interactions. Upper left panel: aphid stylet penetration of the cell wall releases oligogalacturonides (OGs), which induce PAMP/DAMP‐triggered immunity (PTI/DTI) in a process dependent on BRASSINOSTEROID INSENSITIVE 1‐ASSOCIATED RECEPTOR KINASE 1 (BAK1), ENHANCED DISEASE SUSCEPTIBILITY 1 (EDS1), and CPK5/6/11 (data herein). The stabilisation of pattern recognition receptors (PRRs) and coreceptors at the plasma membrane may rely on the deubiquitination (DUB) activity of ASSOCIATED MOLECULE WITH THE SH3 DOMAIN OF STAM (AMSHs; Gravino *et al*., 2024). Upper right panel: the Mp10 effector, introduced by aphids into the cell cytoplasm (Mugford *et al*., 2016), suppresses OG‐induced reactive oxygen species (ROS) (data herein) and flg22‐induced PTI (Bos *et al*., 2010). Mp10 targeting of plant AMSHs is implicated in these processes (Gravino *et al*., 2024). EDS1 is essential for Mp10‐mediated ROS suppression and PRR destabilisation (data herein), acting through an unidentified mechanism (denoted by the double‐sided arrow with an asterisk). Lower left panel: effector‐triggered immunity (ETI) is activated through EDS1, either directly or indirectly, upon recognition of Mp10 and/or its activities by a TNL (Gravino *et al*., 2024; Rao *et al*., 2024). Salicylic acid glucosyltransferase 1 (SGT1) is also required for TNL/ETI activation (Bos *et al*., 2010). Lower right panel: aphids secrete additional effectors, such as cathepsin B proteins (e.g. CathB6), which target EDS1 to suppress ETI (Liu *et al*., 2025). Solid arrows, increased activation; solid blunt‐ended arrows, increased suppression; Dashed arrows, reduced activation; Dashed ‐blunt‐ended arrows, reduced suppression. The model was generated using icons with attribution and licensing details listed in Supporting Information Table S5.

This study sheds light on the intricate dynamics of DTI activation with a specific focus on the roles of OGs as DAMPs and DTI suppression by the aphid saliva CSP effector Mp10 (Fig. 8). We found that OG‐induced DTI effectively limits *M. persicae* colonisation on Arabidopsis and that the key immune components BAK1/BKK1, CPK5/CPK6, GRP3, and EDS1 are pivotal for this DTI response. However, aphids employ diverse strategies to counteract these defences. First, OG production upon wounding is reduced in plants during aphid feeding, indicating that aphids may suppress DAMP release, thereby enhancing their performance. Moreover, the aphid effector Mp10, which is secreted into the cytoplasm of plant cells during the early stages of aphid feeding (Mugford *et al*., 2016), suppresses the plant sensitivity to OGs and flg22 via PRR destabilisation. However, Mp10 also induces ETI, including local and systemic responses, and aphid resistance in an EDS1‐dependent manner. Remarkably, we found that EDS1 is also required for Mp10‐mediated suppressing activities.

OG‐induced DTI against *M. persicae* depends on BAK1/BKK1, GRP3, EDS1, and CPK5/6, while CPK11 promotes plant immunity to aphids. These kinases are members of the CDPK subgroup I (Cheng *et al*., 2002) and are known regulators of defence responses downstream of various PAMPs/DAMPs, contributing to resistance against pathogens, such as *P. syringae* and *B. cinerea* (Boudsocq *et al*., 2010; Dubiella *et al*., 2013; Ma *et al*., 2013; Gravino *et al*., 2015). Activated by calcium, CPK5/6/11 phosphorylate RBOHD via interaction with the lectin receptor‐like kinase LecRK‐IX.2, triggering PTI/DTI responses, including ROS production and ethylene biosynthesis (Boudsocq *et al*., 2010; Kadota *et al*., 2014; Luo *et al*., 2014, 2017; Gravino *et al*., 2015). Aphid performance is unchanged on *cpk5 cpk6* double mutants but improves on *cpk5 cpk6 cpk11* triple mutants, and therefore ROS and ethylene production mediated by CPK5/6/11 may be crucial for aphid resistance. Additionally, BAK1/BKK1 and EDS1 function in related pathways, acting upstream and downstream of calcium elevations, respectively (Ranf *et al*., 2011; Roux *et al*., 2011;Pruitt *et al*., 2021; Tian *et al*., 2021). Consistent with this, *M. persicae* induces calcium elevations in WT plants that require BAK1 and performs better on mutants impaired in ROS production (*rbohd*) and ethylene signalling (*ein2*; Moloi & van der Westhuizen, 2006; Miller *et al*., 2009; Kettles *et al*., 2013; Vincent *et al*., 2017). Together, these findings highlight the central role of BAK1/BKK1, GRP3, EDS1, and CPK5/6/11 in coordinating plant resistance to aphids.

We found that OG release in response to wounding is minimised in plants infested with aphids. One possible explanation is that pectin becomes less susceptible to enzymatic degradation by either aphid salivary enzymes or plant enzymes activated upon wounding. Evidence suggests that plant PME activity increases during aphid feeding on Arabidopsis, leading to blockwise de‐methylesterification and calcium‐mediated cross‐linking of pectin into ‘egg‐box’ structures (Silva‐Sanzana *et al*., 2019). These structural modifications likely hinder the accessibility of pectin‐degrading enzymes, promoting pectin stiffening and reducing OG release (Liners *et al*., 1989; Hocq *et al*., 2017; Wormit & Usadel, 2018). Another consideration is that OGs that are released by epidermal and mesophyll cells may be absorbed by the gel‐like sheath saliva that surrounds the stylets of aphids within the apoplast. Such absorption may limit the diffusion of OGs in the apoplast, prevent their binding to cell‐surface receptors, and reduce their detection using methods herein. This hypothesis is reasonable because the sheath saliva is located in the apoplast where the OGs are released. However, it is important to note that, so far, there is no evidence for OG association with the stylet sheaths of Hemiptera. Nevertheless, our findings suggest that aphids may actively minimise the release of immune‐activating OGs to enhance their performance on host plants.

OG‐induced immunity shares several characteristics with flg22. Mp10 suppresses OG‐ and flg22‐induced immunity (Bos *et al*., 2010;Drurey *et al*., 2019; Gravino *et al*., 2024), which requires BAK1/BKK1, CPK5/CPK6/CPK11, GRP3, and EDS1 (Boudsocq *et al*., 2010; Roux *et al*., 2011; Gramegna *et al*., 2016; Pruitt *et al*., 2021; Tian *et al*., 2021). This overlap further suggests that OGs and flg22 share similar signal transduction elements (Gravino *et al*., 2017). In line with this, flg22 treatment also induced the expression of immune responses that reduce aphid performance (Prince *et al*., 2014a). Nonetheless, we found that Mp10 does not suppress chitin‐induced PTI, in line with previous results (Bos *et al*., 2010). Interestingly, while both OG‐ and flg22‐induced responses require BAK1 (Chinchilla *et al*., 2007; Heese *et al*., 2007; Gravino *et al*., 2017), chitin‐induced responses are BAK1‐independent (Shan *et al*., 2008). This distinction suggests that Mp10 specificity may involve BAK1‐dependent pathways. Future studies should explore whether Mp10 suppression specificity extends to other cell wall‐derived DAMPs, such as cellulose‐ or hemicellulose‐derived fragments (Molina *et al*., 2024), although given that Mp10 also suppresses plant PTI to flg22, we expect that Mp10 may suppress any PRR‐mediated response that involves BAK1.

The aphid effector Mp10 exhibits dual roles in plant immunity, suppressing and inducing responses, a characteristic shared with pathogen effectors like AvrPto, AvrPtoB, and HopB1. The latter three effectors act by modulating FLS2 and other cell‐surface receptor kinases (RKs). AvrPto inhibits their kinase activity (Xiang *et al*., 2008), while AvrPtoB and HopB1 promote the degradation of these receptors via ubiquitination or proteolytic cleavage (Goehre *et al*., 2008; Gimenez‐Ibanez *et al*., 2009; Li *et al*., 2016), and the activities of these effectors are detected by intracellular immune receptors, leading to ETI (Mathieu *et al*., 2014; Schulze *et al*., 2022). Similarly, Mp10 destabilises immune‐related RKs, including FLS2 and FER, in a process that involves interaction of Mp10 with AMSH proteins – key deubiquitinating enzymes essential for protein trafficking – thereby rendering plants insensitive to PAMPs (Gravino *et al*., 2024). Mp10 may also target putative OG receptors. However, the identity of these receptors remains elusive, as studies using WAK quintuple loss‐of‐function mutants showed that WAKs are entirely dispensable for the plant response to OGs (Herold *et al*., 2024) or that their absence might be compensated by redundant WAK‐like or analogous receptors (Blaschek, 2024), leaving this hypothesis for future investigation. Given that depletion of RKs, including BAK1 and BKK1, from the plasma membrane is known to activate ETI via the EDS1 pathway (Gao *et al*., 2017; Wu *et al*., 2020; Schulze *et al*., 2022), our finding that Mp10 simultaneously suppresses PTI and triggers ETI is not unexpected. We also observed that aphids perform worse on the *bak1‐5 bkk1‐1* double mutant, in contrast to the role of BAK1 and BKK1 as positive regulators of plant immunity against bacterial and oomycete pathogens (Roux *et al*., 2011). Since Mp10 destabilises cell‐surface RKs and activates ETI via EDS1, the reduced aphid colonisation on *bak1‐5 bkk1‐1* could be explained by the hypothetical destabilisation of BAK1‐5, or related SOMATIC EMBRYOGENESIS RECEPTOR KINASE (SERK) family members (Meng *et al*., 2015), during aphid feeding. This may in turn trigger ETI and enhance aphid resistance, similar to what has been observed for the Pseudomonas effector HopB1 (Li *et al*., 2016; Wu *et al*., 2020; Schulze *et al*., 2022).

We found that Mp10‐induced immunity exhibits differential dependency on EDS1, ADR1, NRG1, and SA. This includes a local transient expression of Mp10 inducing a weak response, characterised by chlorosis confined to the infiltration area, which is dependent on EDS1 and ADR1. This response does not require NRG1, which induces a strong immune activation, such as cell death (Lapin *et al*., 2019), in line with the absence of a clear cell death response to Mp10 in this and earlier studies (Bos *et al*., 2010; Rao *et al*., 2024). Additionally, the dependence of the local response on SA is consistent with the established role of EDS1 in mediating both SA‐dependent and SA‐independent immune pathways (Bartsch *et al*., 2006; Straus *et al*., 2010; Cui *et al*., 2017).

Mp10 triggers also a systemic effect in plants, as evidenced by a dwarfing phenotype of plants, and this phenotype is dependent on NRG1, as well as EDS1 and SA. Since EDS1 promotes ETI downstream of TIR‐NBS‐LRR (TNL) immune receptors (Aarts *et al*., 1998; Feys *et al*., 2001; Sun *et al*., 2021), our results suggest that Mp10 itself and/or its activities of perturbing cell‐surface receptor kinases (Gravino *et al*., 2024) may be recognised/guarded by TNL immune receptors (Greenwood & Williams, 2022). Mp10 is a CSP that is present in other sap‐feeding insects, including whiteflies, leafhoppers, and planthoppers (Drurey *et al*., 2019; Gravino *et al*., 2024). Recent evidence suggests that these CSPs are recognised by the *N. benthamiana* TNL‐CJID (C‐terminal jelly roll/Ig‐like domain) protein RCSP, which also requires EDS1 for inducing chlorosis and dwarfing responses (Rao *et al*., 2024). More research is needed to assess whether this receptor is involved in *M. persicae* Mp10‐induced chlorosis and dwarfing responses reported herein.

Our results reveal that EDS1 is involved in Mp10‐mediated immune‐suppressing activities. EDS1 is a conserved immune regulator essential for ETI and its crosstalk with PTI. In Arabidopsis, the EDS1‐PAD4‐ADR1 complex is linked to PRR complexes via SOBIR1 for downstream PTI signalling (Pruitt *et al*., 2021; Tian *et al*., 2021). An EDS1 pathway also monitors receptor kinase homeostasis involved in PTI and activates ETI when perturbations occur (Schulze *et al*., 2022; Yang *et al*., 2022; Yu *et al*., 2023). We found that Mp10 suppresses flg22‐triggered PTI and destabilises FLS2, consistent with previous reports (Gravino *et al*., 2024). However, in the absence of EDS1, Mp10 no longer affects FLS2 stability or downstream signalling. The mechanism by which EDS1 contributes to Mp10 suppressive activity is unclear. Given the association of EDS1 with membrane proteins (Pruitt *et al*., 2021), this Mp10 activity may be influenced by its interactions with AMSH proteins, which are involved in membrane protein trafficking (Gravino *et al*., 2024), and whose functions could vary depending on the presence or absence of EDS1. Notably, OG‐mediated DTI depends on EDS1 and is suppressed by Mp10. In Arabidopsis *eds1‐2* × Mp10 cross lines, OG‐mediated DTI immunity to aphids is restored, whereas it is absent in *eds1‐2*, indicating that Mp10 compensates for the DTI response or fails to suppress DTI in the *eds1‐2* mutant background. This suggests a complex interplay between Mp10 and EDS1 in regulating OG‐inducible DTI. It is possible that Mp10 and EDS1 act in a mutually repressive manner or could inversely regulate distinct OG‐inducible DTI pathways, activating one while suppressing another. Given the likelihood of multiple, redundant OG perception and signalling complexes (Gravino *et al*., 2017; Blaschek, 2024), EDS1 absence may be compensated by alternative OG receptors operating independently of EDS1, but only in the presence of Mp10, enhancing plant resilience. In this hypothetical scenario, in the absence of Mp10, OG‐induced DTI depends on an EDS1‐dependent receptor. However, when Mp10 is present, it may target or bypass the EDS1‐dependent pathway, enabling the activation of alternative OG receptors that function independently of EDS1. Future biochemical studies are needed to elucidate how Mp10 is compensating for the loss of EDS1 and the link between Mp10, OG suppression, and EDS1 dependence. Nevertheless, our findings identify EDS1 as a key player in Mp10's dual role: suppressing DTI/PTI responses while also eliciting ETI.

We have shown that transcripts corresponding to *Mp10* are readily detected in our Arabidopsis DEX‐inducible Mp10 lines following DEX treatment, confirming that the transgene is transcriptionally active. Moreover, induction of these lines results in phenotypes typically associated with Mp10 expression, including chlorosis and stunted growth, consistent with previous observations in *N. benthamiana* plants transiently overexpressing Mp10 (Bos *et al*., 2010; Rao *et al*., 2024). However, we were unable to detect Mp10 proteins in these lines using our specific Mp10 antibodies. By contrast, the same expression cassette used to generate the Arabidopsis lines leads to detectable Mp10 proteins when transiently overexpressed in *N. benthamiana* leaves via *A. tumefaciens*‐mediated transformation. One possible explanation is that Mp10 proteins are unstable and therefore accumulate at levels below the detection threshold in the Arabidopsis inducible system. This is consistent with our recent findings that Mp10 associates with AMSH proteins, which function in the endosomal protein degradation pathway (Gravino *et al*., 2024). It is therefore plausible that Mp10 is actively targeted for degradation through this pathway. Further investigation will be needed to test this possibility.

We did not observe Mp10 affecting *A. tumefaciens*‐mediated transient protein expression in *N. benthamiana*, as reported previously (Rodriguez *et al*., 2014). Differences between the studies may reflect protein sample overloading in the gel, variability in band intensities of the loading controls, absence of quantitative analyses and normalisation, and lack of clarity on whether experimental proteins with Mp10 or vector control were expressed side by side in the same leaf in the earlier work. While Rodriguez and colleagues proposed that Mp10 might suppress *A. tumefaciens*‐mediated transcription, this mechanism was not explored in detail. Our data do not support a general suppression of *A. tumefaciens*‐mediated transient protein expression by Mp10.

Aphids are highly specialised feeders, and their feeding actions affect only a few cells in close proximity to their stylets (Tjallingii & Esch, 1993). This localised feeding strategy is evidenced by minimal calcium release upon aphid stylet penetrations (Vincent *et al*., 2017; Joyce, 2023) compared with the robust calcium release triggered by piercing‐sucking thrips (Joyce, 2023) or leaf‐chewing caterpillars (Toyota *et al*., 2018). Moreover, aphids deliver small quantities of effectors into plant cells, possibly in a sequential manner and tailored to specific cell types (Sanchez‐Garrido *et al*., 2022). By contrast, experimental applications of OGs and Mp10 affect entire leaves or plants. Thus, the impacts of OGs and Mp10 are likely to be more nuanced and temporally refined within the aphid feeding site, where additional factors shape the final outcomes of plant–aphid interactions. In line with this is the finding that Mp10 is present in the acrostyle at the tip of the stylets from where it is likely to be delivered into the cell cytoplasm during the brief salivation periods in the probing phase (Brault *et al*., 2010; Deshoux *et al*., 2022), as evidenced by the presence of Mp10 in the cytoplasm of cells near aphid stylets (Mugford *et al*., 2016). Therefore, Mp10 is likely to be present at the early feeding stages, at the time when aphids just have started their probing behaviour, when suppression of DTI/PTI responses is needed. Aphids also deliver other effectors, including cathepsin B‐like proteins (CathBs), such as CathB6, which interacts with EDS1, recruits it to processing bodies, and suppresses EDS1‐mediated immunity (Liu *et al*., 2025).

This study, together with previous research on aphid effectors and plant responses, contributes to a comprehensive model encompassing all key players involved (Fig. 8). These findings underscore the dynamic interplay between plant defences and aphid countermeasures, highlighting the critical role of OGs in activating DTI and the adaptive strategies aphids employ, such as suppressing OG production and deploying effectors like Mp10, to modulate host defences. This knowledge will contribute to devising durable and sustainable strategies to control aphids and potentially other sap‐sucking insects and the viruses they transmit.

## Competing interests

None declared.

## Author contributions

MG, GDL and SAH acquired funding for the research. SAH managed the project and staff. MG, STM, DP, JJ, FC, GDL and SAH planned and designed research. MG, STM, DP and JJ performed experiments. MG, STM, DP and JJ collected and analysed data. MG, STM, DP, JJ, FC, GDL and SAH interpreted data. MG, STM, CD and DCP contributed new plant material. MG, DP, FC, GDL and SAH wrote the original draft of the paper. All authors reviewed, edited, and approved the final manuscript.

## Disclaimer

The New Phytologist Foundation remains neutral with regard to jurisdictional claims in maps and in any institutional affiliations.

## Supporting information


**Dataset S1** Source data for figures supporting the findings of this study.

**Fig. S1** Genotyping of the *Arabidopsis thaliana* transgenic lines used in this work.
**Fig. S2** Dose–response analysis of β‐oestradiol in *Arabidopsis thaliana XVE:OGM* transgenic and Col‐0 wild‐type plants.
**Fig. S3** Content of oligogalacturonides (OGs) in aphid‐infested and control *Arabidopsis thaliana* leaves.
**Fig. S4** High‐performance anion‐exchange chromatography (HPAEC) with pulsed amperometric detector (PAD) profiles of oligogalacturonides (OGs) extracted with or without sodium sulphite from aphid‐infested and control *Arabidopsis thaliana* leaves.
**Fig. S5** Characterisation of *Mp10* transcript levels in response to dexamethasone (DEX) in *Arabidopsis thaliana* DEX‐inducible Mp10 transgenic lines.
**Fig. S6** Analysis of the oligogalacturonide (OG)‐triggered immunity in the *Arabidopsis thaliana* dexamethasone (DEX)‐inducible Mp10 lines in the absence of DEX induction.
**Fig. S7** The aphid effector Mp10 suppresses reactive oxygen species (ROS) bursts triggered by bacterial flg22, but not fungal chitin, in *Arabidopsis thaliana*.
**Fig. S8** Additional analyses of the effector‐triggered immunity (ETI)‐like responses induced by the aphid effector Mp10 in *Nicotiana benthamiana* NahG and *nrg1* plants.
**Fig. S9** The aphid effector Mp10 promotes EDS1‐dependent defence activation in *Arabidopsis thaliana*.
**Fig. S10** EDS1 is required for Mp10‐mediated suppression of the reactive oxygen species (ROS) burst induced by bacterial flg22 in *Nicotiana benthamiana*.
**Fig. S11** Source western blot data for Fig. 7.
**Methods S1** Quantification of DAB staining in *Arabidopsis thaliana* leaves using the Fiji plugin Colour Deconvolution 2.
**Table S1** List of *Arabidopsis thaliana* transgenic lines used in this study.
**Table S2** List of primer sequences used in this study.
**Table S3** *Arabidopsis thaliana* genes and elements involved in both oligogalacturonide (OG) signalling and immunity to *Myzus persicae*.
**Table S4** *Post hoc* comparisons – genotype ✻ treatment, relative to Fig. 6.
**Table S5** List of icons, including attribution and licence, used to generate the model in Fig. 8.
Please note: Wiley is not responsible for the content or functionality of any Supporting Information supplied by the authors. Any queries (other than missing material) should be directed to the *New Phytologist* Central Office.

## Data Availability

The source data of the figures that support the findings of this study are available in Fig. S11 and Dataset S1.
