# Supplementary material for: Aphid effector Mp10 balances immune suppression and defence activation through EDS1‐dependent modulation of plant DAMP responses
Source: New Phytol. 2025 Jul 30;248(2):913–35. doi: 10.1111/nph.70419 (PMC12445834; doi:10.1111/nph.70419)
Supplement: Supplementary file 2 — Fig. S1 Genotyping of the Arabidopsis thaliana transgenic lines used in this work. Fig. S2 Dose–response analysis of β‐oestradiol in Arabidopsis thaliana XVE:OGM transgenic and Col‐0 wild‐type plants. Fig. S3 Content of oligogalacturonides (OGs) in aphid‐infested and control Arabidopsis thaliana leaves. Fig. S4 High‐performance anion‐exchange chromatography (HPAEC) with pulsed amperometric detector (PAD) profiles of oligogalacturonides (OGs) extracted with or without sodium sulphite from aphid‐infested and control Arabidopsis thaliana leaves. Fig. S5 Characterisation of Mp10 transcript levels in response to dexamethasone (DEX) in Arabidopsis thaliana DEX‐inducible Mp10 transgenic lines. Fig. S6 Analysis of the oligogalacturonide (OG)‐triggered immunity in the Arabidopsis thaliana dexamethasone (DEX)‐inducible Mp10 lines in the absence of DEX induction. Fig. S7 The aphid effector Mp10 suppresses reactive oxygen species (ROS) bursts triggered by bacterial flg22, but not fungal chitin, in Arabidopsis thaliana. Fig. S8 Additional analyses of the effector‐triggered immunity (ETI)‐like responses induced by the aphid effector Mp10 in Nicotiana benthamiana NahG and nrg1 plants. Fig. S9 The aphid effector Mp10 promotes EDS1‐dependent defence activation in Arabidopsis thaliana. Fig. S10 EDS1 is required for Mp10‐mediated suppression of the reactive oxygen species (ROS) burst induced by bacterial flg22 in Nicotiana benthamiana. Fig. S11 Source western blot data for Fig. 7. Methods S1 Quantification of DAB staining in Arabidopsis thaliana leaves using the Fiji plugin Colour Deconvolution 2. Table S1 List of Arabidopsis thaliana transgenic lines used in this study. Table S2 List of primer sequences used in this study. Table S3 Arabidopsis thaliana genes and elements involved in both oligogalacturonide (OG) signalling and immunity to Myzus persicae. Table S4 Post hoc comparisons – genotype ✻ treatment, relative to Fig. 6. Table S5 List of icons, including attribution and licence, [file NPH-248-913-s002.docx]

New Phytologist Supporting Information

Article title: **Aphid effector Mp10 balances immune suppression and defence activation through EDS1-dependent modulation of plant DAMP responses**

Authors: Matteo Gravino, Sam T. Mugford, Daniela Pontiggia, Joshua Joyce, Claire Drurey, David C. Prince, Felice Cervone, Giulia De Lorenzo, Saskia A. Hogenhout

Article acceptance date: 04 July 2025

The following Supporting Information is available for this article:

**Fig. S1** Genotyping of the *Arabidopsis thaliana* transgenic lines used in this work

**Fig. S2** Dose-response analysis of β-oestradiol in *Arabidopsis thaliana* *XVE:OGM* transgenic and Col-0 wild-type plants

**Fig. S3** Content of oligogalacturonides (OGs) in aphid-infested and control *Arabidopsis thaliana* leaves

**Fig. S4** High-performance anion-exchange chromatography (HPAEC) with pulsed amperometric detector (PAD) profiles of oligogalacturonides (OGs) extracted with or without sodium sulphite from aphid-infested and control *Arabidopsis thaliana* leaves

**Fig. S5** Characterisation of *Mp10* transcript levels in response to dexamethasone (DEX) in *Arabidopsis thaliana* DEX-inducible Mp10 transgenic lines

**Fig. S6** Analysis of the oligogalacturonide (OG)-triggered immunity in the *Arabidopsis thaliana* dexamethasone (DEX)-inducible Mp10 lines in the absence of DEX induction

**Fig. S7** The aphid effector Mp10 suppresses reactive oxygen species (ROS) bursts triggered by bacterial flg22, but not fungal chitin, in *Arabidopsis thaliana*

**Fig. S8** Additional analyses of the effector-triggered immunity (ETI)-like responses induced by the aphid effector Mp10 in *Nicotiana benthamiana* NahG and *nrg1* plants

**Fig. S9** The aphid effector Mp10 promotes EDS1-dependent defense activation in *Arabidopsis thaliana*

**Fig. S10** EDS1 is required for Mp10-mediated suppression of the reactive oxygen species (ROS) burst induced by bacterial flg22 in *Nicotiana benthamiana*

**Fig. S11** Source western blot data for Fig. 7

**Table S1** List of *Arabidopsis thaliana* transgenic lines used in this study

**Table S2** List of primer sequences used in this study

**Table S3** *Arabidopsis thaliana* genes and elements involved in both oligogalacturonide (OG) signalling and immunity to *Myzus persicae*

**Table S4** Post hoc comparisons - genotype ✻ treatment, relative to Fig. 6

**Table S5** List of icons, including attribution and license, used to generate the model in Fig. 8

**Method S1** Quantification of DAB staining in *Arabidopsis thaliana* leaves using the Fiji plugin Colour Deconvolution 2

Separate additional excel file:

**Dataset S1** Source data for figures supporting the findings of this study

**Fig. S1** Genotyping of the *Arabidopsis thaliana* transgenic lines used in this work. (a) Genotyping of the *XVE:OGM* and *pPR1:OGM* transgenic plants by PCR using genomic DNA (gDNA) as a template. (b, c, d) Transcript analyses in *bak1-5 bkk1-1*, *cpk5 cpk6*, *cpk5 cpk6 cpk11*, and *grp3* mutants, and in *GRP3*-OE transgenic plants, compared to Col-0 wild type (WT), by reverse transcriptase (RT)-PCR using complementary DNA (cDNA) as a template. In (b), asterisks indicate the RT-PCR products of the *bak1-5 bkk1-1* mutant and Col-0 WT, as a control, that were sequenced, confirming the point mutation TGC>TAC resulting in the expected Cys408Tyr amino acid substitution indicated by the solid arrow on top of the right panel in the coding DNA sequence (CDS) of transcripts of the *bak1-5* allele. Nucleotide alignment was conducted using MEGA version X (Kumar *et al.*, 2018). (e) Analysis of the *EDS1* deletion (top panel) in the *eds1-2* mutant, *eds1-2* x Mp10 #7-5, and *eds1-2* x Mp10 #9-5 crosses, compared to Mp10 transgenic lines (#7-5 and #9-5) by PCR using gDNA as a template. The bottom panel confirms the presence of the *Mp10* transgene in the Mp10 and *eds1-2* x Mp10 lines but not in the *eds1-2* mutant. (a, b, c, d, e) Gene-specific primers used for genotyping are listed in Supporting Information Table S2.

**Fig. S2** Dose-response analysis of β-oestradiol in *Arabidopsis thaliana* *XVE:OGM* transgenic and Col-0 wild-type plants. (a) One leaf from 4-week (w)-old Arabidopsis *XVE:OGM* transgenic plants was infiltrated with dimethyl sulfoxide (DMSO) or 0.5, 5, or 50 μg ml^-1^ β-oestradiol (dissolved in DMSO) (x-axis). Transcript levels of *OG-machine* (*OGM*), *BERBERINE BRIDGE ENZYME-LIKE 3* (*BBE3*/*FOX1*/*RET-OX*), and *FLG22-INDUCED RECEPTOR-LIKE KINASE 1* (*FRK1*) relative to *GLYCERALDEHYDE 3-PHOSPHATE DEHYDROGENASE* (*GAPDH*) (y-axis) were measured by quantitative reverse transcriptase (qRT)-PCR 3 days post infiltration (dpi). Bars are means ± SE. n = 3 replicates shown as black dots. (b) One leaf from 4-w-old Arabidopsis Col-0 wild type (WT) was infiltrated with DMSO or 0.5, 5, or 50 μg ml^-1^ β-oestradiol (dissolved in DMSO) (x-axis). 3 dpi, one 6-d-old asexually reproducing *M. persicae* adult female was caged on the infiltrated leaf. After 10 d, the number of individual aphids (adults + nymphs) within the cage was counted to obtain the aphid colony size (y-axis). Results are shown as means ± SE. n = 7 replicates shown as black dots. (a, b) Asterisks indicate significant differences between DMSO- and β-oestradiol-treated samples as determined by one-way ANOVA with post-hoc Tukey HSD test (*, *P* < 0.05; **, *P* < 0.01; ***, *P* < 0.001; ns, not significant).

**Fig. S3** Content oligogalacturonides (OGs) in aphid-infested and control *Arabidopsis thaliana* leaves. (a) Content of common peaks, including different OG oligomers with degree of polymerisation (DP) 6-15, in the chromatograms of controls and aphid-infested leaves for 6 h. The x-axis shows the peak number (in order of elution): peaks 1 to 5, unknown peaks (not highlighted), cannot be clearly identified as pectic fragments; peaks 6 to 15 (highlighted in grey) correspond to OG DP6 to 15. The y-axis shows the peak area (nC min), normalised using the compositional data normalisation (CoDA) method. Bars are means ± SE. Observations are indicated with dots for experiment (exp) 1 or triangles for exp 2. n = 3 replicates in each experiment. (b) Experiment design to detect OGs during late aphid feeding. Leaves from 4-week-old *A. thaliana* Col-0 wild-type (WT) plants were caged with two 6-d-old asexually reproducing *M. persicae* adult females (red arrowheads, panel I) or without aphids (empty cage), as a control. 7 or 9 days post infestation (dpi), leaves were excised and, after removing all aphids, sliced into strips (panel II). Leaf strips were incubated with strong chelating agents supplemented with sodium sulphite (ChA solution) for 16 h (panel III). The leaf diffusates in incubation medium were analysed by high-performance anion-exchange chromatography (HPAEC) with a pulsed amperometric detector (PAD) (panel IV); this figure was created in BioRender (BioRender.com/EI25PBPRUZ). (c) Aphid colony size (y-axis) inside clip cages after 7 and 9 dpi (x-axis). Bars are means ± SE. Observations are indicated with dots for experiment (exp) 1 or triangles for exp 2. n = 3 replicates in each experiment. (d) Chromatographic analysis of leaf diffusates in ChA solution obtained from leaf strips of plants infested with aphids or non-infested, as a control, for 7 and 9 d. The numbers on top of the graph indicate the degree of polymerisation (DP) of different OG oligomers and refer to the corresponding peaks below. The x-axis shows the retention time measured in min. The y-axis shows the detector response measured in nanocoulombs (nC). The bottom panel shows the profile of 2.5 μg of standard OGs enriched in the DP range 10-15. For each treatment, the result of one representative replicate out of three performed is shown. (e) Content of common peaks, including different OG oligomers (DP3, DP6-15), in the chromatograms of controls and aphid-infested leaves for 7 and 9 d. The x-axis shows the peak number (in order of elution): peaks 1 to 9 and 1 to 5, unknown (unk.) peaks (not highlighted), cannot be clearly identified as pectic fragments in experiments 1 and 2, respectively; peaks 10 to 19 and 7 to 16 correspond to OG DP6 to 15 in experiments 1 and 2, respectively; peak 6 in experiment 2 corresponds to OG DP3. The y-axis shows the peak area (nC min) of the common peaks, normalised using the compositional data normalisation (CoDA) method. Bars are means ± SE. Observations are indicated with dots. n = 3 replicates in each experiment. In (a, e), asterisks indicate significant differences between aphid-infested leaves and control samples as determined by one-way ANOVA with post-hoc Tukey HSD test or Student's *t* test, respectively (*, *P* < 0.05; **, *P* < 0.01; ***, *P* < 0.001). This figure was created with BioRender.com ([https://BioRender.com](https://eur02.safelinks.protection.outlook.com/?url=https%3A%2F%2Fbiorender.com%2F&data=05%7C02%7Cr.mordue%40lancaster.ac.uk%7C633fb3a756ca41bc17a008ddc2e9d53c%7C9c9bcd11977a4e9ca9a0bc734090164a%7C0%7C0%7C638881033068673458%7CUnknown%7CTWFpbGZsb3d8eyJFbXB0eU1hcGkiOnRydWUsIlYiOiIwLjAuMDAwMCIsIlAiOiJXaW4zMiIsIkFOIjoiTWFpbCIsIldUIjoyfQ%3D%3D%7C0%7C%7C%7C&sdata=BhYgQ7zLz2sUco13Li85THLbHeC9IRIbpxuyABKmjLk%3D&reserved=0)).

**Fig. S4** High-performance anion-exchange chromatography (HPAEC) with pulsed amperometric detector (PAD) profiles of oligogalacturonides (OGs) extracted with or without sodium sulphite from aphid-infested and control *Arabidopsis thaliana* leaves. Leaf strips were obtained from 4-week-old *A. thaliana* Col-0 wild-type (WT) plants infested with aphids or non-infested, as a control, for 7 or 9 days. Leaf strips were incubated for 16 h in strong chelating agents (ChA solution, i.e., 50 mM ammonium acetate pH 5.0, 50 mM CDTA, 50 mM ammonium oxalate) supplemented with 10 mM sodium sulphite (ChA buffer + sodium sulphite), to inhibit the activity of OG oxidases (Benedetti *et al.*, 2017) or without sodium sulphite (ChA buffer) to detect eventual presence of oxidised OGs (Benedetti *et al.*, 2018). Leaf diffusates in the buffers were then analysed by HPAEC‐PAD. (a,b) Experiment 1 and 2, performed with ChA buffer + sodium sulfite. (c,d) Experiment 1 and 2, performed with ChA buffer. (a, b, c, d) The numbers on top of the graphs indicate the degree of polymerisation (DP) of different OG oligomers and refer to the corresponding peaks below. The x-axis shows the retention time for different OG oligomers measured in min. The y-axis shows the detector response for different OG oligomers measured in nanocoulombs (nC). The bottom panels show the profile of 2.5 μg of standard OGs enriched in the DP range 10-15. For each experiment, the result of one representative replicate out of three performed is shown.

**Fig. S5** Characterisation of *Mp10* transcript levels in response to dexamethasone (DEX) in *Arabidopsis thaliana* DEX-inducible Mp10 transgenic lines. (a, b) Transcript levels of *Mp10* relative to *GLYCERALDEHYDE 3-PHOSPHATE DEHYDROGENASE* (*GAPDH*) and *UBIQUITIN 5* (*UBQ5*) (y-axis) were measured by quantitative reverse transcriptase (qRT)-PCR in leaves of 4-week-old *A. thaliana* Mp10 transgenic lines (#7-5 and #9-5) (x-axis) at 24, 48, 72, and 144 h after spraying with 20 μM DEX and at 144 h after spraying with dimethyl sulfoxide (DMSO), as a control (a), and at 72 h after spraying with DMSO or 10, 5, 2.5, or 1.25 μM DEX (b). Results are shown as mean ± SE. n = 2 biological repeats.

**Fig. S6** Analysis of the oligogalacturonide (OG)-triggered immunity in the *Arabidopsis thaliana* dexamethasone (DEX)-inducible Mp10 lines in the absence of DEX induction. In (a, b, c), 3- to 4-week (w)-old plants of *A. thaliana* Col-0 wild type (WT) and DEX-inducible Mp10 transgenic lines (#7-5 and #9-5) were sprayed with dimethyl sulfoxide (DMSO) 3 d before treatment with 200 μg ml^-1^ OG and H_2_O as a control. (a) Reactive oxygen species (ROS) production measured in leaf discs over a period of 30 min after elicitor treatment. Solid line, mean ± SE. *n*= 8 and 16 leaf discs treated with H_2_O and OGs, respectively. The x-axis shows the time in min after elicitor treatment. The y-axis shows the photon count. (b) Total ROS production over a period of 30 min after elicitor treatment. *n*= 8 and 16 leaf discs treated with H_2_O and OGs, respectively. The y-axis shows the total photon count. The x-axis shows the plant genotype. (c) Transcript levels of *FLG22-INDUCED RECEPTOR-LIKE KINASE 1* (*FRK1*), *CYTOCHROME P450, FAMILY 81, SUBFAMILY F, POLYPEPTIDE 2* (*CYP81F2*), *PHYTOALEXIN DEFICIENT 3* (*PAD3*), and *PHYTOALEXIN DEFICIENT 4* (*PAD4*) relative to *GLYCERALDEHYDE 3-PHOSPHATE DEHYDROGENASE* (*GAPDH*) and *UBIQUITIN 5* (*UBQ5*) (y-axis) measured 2 h after elicitor infiltration. *n* = 3 in each experiment. In (b, c), boxplots show the median, the 25^th^ and 75^th^ percentiles, the most extreme data points (whiskers’ extensions), and the observations as black-filled circles in experiment (exp) 1, triangles in exp 2, or squares in exp 3. Data points above the top whisker or below the bottom whisker are outliers. Ns, not significant differences between samples as determined by one-way ANOVA or two-way ANOVA with interaction (genotype:treatment) and post-hoc Tukey HSD test.

**Fig. S7** The aphid effector Mp10 suppresses reactive oxygen species (ROS) bursts triggered by bacterial flg22, but not fungal chitin, in *Arabidopsis thaliana*. (a) Total ROS production over a period of 8 h after flg22 treatment. (b) Total ROS production over a period of 5 h after chitin treatment. In (a, b), 3- to 4-week (w)-old plants of *A. thaliana* Col-0 wild type (WT) and dexamethasone (DEX)-inducible Mp10 transgenic lines (#7-5 and #9-5) were sprayed with 1.25 μM DEX and, 3 d later, treated with H_2_O, 100 nM flg22, or 500 μg ml^-1^ chitin, dissolved in water. The y-axis shows the total photon count. The x-axis shows the plant genotype. Boxplots show the median, the 25^th^ and 75^th^ percentiles, the most extreme data points (whiskers’ extensions), and the observations as black-filled circles for experiment (exp) 1 or triangles for exp 2. Data points above the top whisker or below the bottom whisker are outliers. *n*= 8 and 16 leaf discs treated with H_2_O and flg22/chitin, respectively, in each experiment. Asterisks indicate significant differences between Col-0 WT and Mp10 transgenic lines treated with flg22 or chitin, as determined by one-way ANOVA with post-hoc Tukey HSD test (***, *P* < 0.001; ns, not significant).

**Fig. S8** Additional analyses of the effector-triggered immunity (ETI)-like responses induced by the aphid effector Mp10 in *Nicotiana benthamiana* NahG and *nrg1* plants. (a) Plant height and systemic chlorosis measurements in wild-type (WT) and mutant plants agroinfiltrated with Flag-Mp10. The x-axes show the plant genotype. The y-axes show the plant height, defined as the distance between the apex (tip) and the base (at the soil surface) of the stem measured in centimetres (cm) (left panel), and systemic chlorosis, quantified as the average of the red and green colour components in the systemic leaves (right panel). Boxplots show the median, the 25^th^ and 75^th^ percentiles, the most extreme data points (whiskers’ extensions), and the observations as circles in experiment (exp) A or triangles in exp B. Data points above the top whisker or below the bottom whisker are outliers. *n* = 3 in each experiment. Asterisks indicate significant differences between control (WT) and mutant plants as determined by one-way ANOVA with post-hoc Tukey HSD test (***, *P* < 0.001). (b) Representative plants of WT and indicated mutants showing systemic chlorosis and dwarfism (+) or not (-) after agroinfiltration with Flag-Mp10 on one leaf side. White arrowheads indicate systemic leaves with the most severe symptoms, observed only in WT plants. Pictures were taken at 14 days post-infiltration (dpi). Scale bar = 1 cm.

**Fig. S9** The aphid effector Mp10 promotes EDS1-dependent defense activation in *Arabidopsis thaliana*. (a, b, c, d, e, f) Analysis of chlorophyll content and fresh weight in *A. thaliana* plants pretreated with 1.25 μM dexamethasone (DEX) and grown under (a, b, c) normal conditions or (d, e, f) continuous darkness for 5 d. In (a, d), the y-axis shows the micromolar (μmol) chlorophyll content per gram (g) of fresh weight (FW). In (b, e), the y-axis shows the fresh weight of the rosette measured in mg. (c) Representative pictures showing plant size as well as symptoms of chlorosis and senescence, as indicated by white arrowheads, only in the *A. thaliana* Mp10 #7-5 line but not in other plants. (f) Representative pictures showing plant size as well as increased symptoms of chlorosis and senescence in *A. thaliana* Mp10 #9-5 line compared to Col-0, *eds1-2*, and *eds1-2* x Mp10 #9-5. (g, h, i, j) Quantification of DAB staining, indicative of *in situ* production of hydrogen peroxide (H_2_O_2_), in *A. thaliana* plants pretreated with DEX and grown under (g, i) normal conditions or (h, j) continuous darkness for 3 d. In (g, h), the y-axis shows the percentage (%) of the leaf area stained with DAB. (i) Representative leaves showing accumulation of H_2_O_2_ only in Mp10 #7-5. (j) Representative leaves showing increased accumulation of H_2_O_2_ in Mp10 #9-5 compared to controls and *eds1-2* x Mp10 #9-5. In (a, b, d, e, g, h), the x-axis shows the plant genotype. Boxplots show the median, the 25^th^ and 75^th^ percentiles, the most extreme data points (whiskers’ extensions), and the observations as black-filled circles for experiment (exp) 1 or triangles for exp 2. Data points above the top whisker or below the bottom whisker are outliers. n ≥ 3 samples in each experiment. Different letters indicate significant differences between samples as determined by one-way ANOVA with post-hoc Tukey HSD test (*P* < 0.001, *P* < 0.001, *P* < 0.05, *P* < 0.01, *P* < 0.001, *P* < 0.001, respectively). In (c, f, i, j), scale bars = 1 cm.

**Fig. S10** EDS1 is required for Mp10-mediated suppression of the reactive oxygen species (ROS) burst induced by bacterial flg22 in *Nicotiana benthamiana*. (a) Total ROS production over 1 h following treatment with 100 nM flg22 or H₂O (control) in *N. benthamiana* WT or *eds1* mutant leaf discs agroinfiltrated with eGFP alone (white boxes) or eGFP-Mp10 (grey boxes). Y-axis: total photon count; X-axis: plant genotype. Boxplots display the median, 25^th^ and 75^th^ percentiles, whiskers indicating extreme data points, and observations as circles coloured in yellow for experiment (exp) 1, blue for exp 2, or red for exp 3. Data points above the top whisker or below the bottom whisker are outliers. *n* ≥ 3 and *n* ≥ 15 leaf discs per H₂O and flg22 treatment, respectively, per experiment. Asterisks indicate significant differences (one-way ANOVA, Tukey HSD; ***, P < 0.001; ns, not significant).

**Fig. S11** Source western blot data for Fig. 7. Regions of interest are highlighted with dotted blue rectangles corresponding to (a) Fig. 7b and (b) Fig. 7d. Lanes are numbered from 1 to 15, with sample identities indicated in legends on the right. Antibodies used for detection are specified in the bottom left corner of each blot. Ponceau S staining is shown as a loading control.

**Table S1** List of *Arabidopsis thaliana* transgenic lines used in this study

| **Line** | **Locus** | **Description** | **Reference** | **Genotyping** |
| --- | --- | --- | --- | --- |
| *bak1-5 bkk1-1* | *At4g33430*  *At2g13790* | Double mutant generated by cross of *bak1-5* (C408Y) and *bkk1-1* (SALK_057955) in Col-0 background | (Schwessinger *et al.*, 2011) | RT-PCR (*bkk1-1*); Sequencing of RT-PCR products (*bak1-5*) |
| *cpk5 cpk6* | *At4g35310*  *At2g17290* | Double insertional mutant generated by cross of *cpk5* (sail_657C06) and *cpk6* (salk_025460) in Col-0 background | (Gravino *et al.*, 2015) | RT-PCR |
| *cpk5 cpk6 cpk11* | *At4g35310 At2g17290*  *At1g35670* | Triple insertional mutant generated by cross of *cpk5* (sail_657C06), *cpk6* (salk_025460), and *cpk11* (salk_054495) in Col-0 background | (Boudsocq *et al.*, 2010) | RT-PCR |
| *grp3* | *At2g05520* | Insertional mutant (SALK_084685.46.60) in Col-0 background | (Gramegna *et al.*, 2016) | RT-PCR |
| *GRP3-OE #16-4* |  | Transgenic line expressing GRP3 (*At2g05520*)-RFP fusion under control of the CaMV 35S promoter in Col-0 background | (Gramegna *et al.*, 2016) | RT-PCR |
| *eds1-2* | *At3g48090* | Deletion mutant in Col-0 background | (Bartsch *et al.*, 2006) | PCR |
| *XVE:OGM* |  | Transgenic line expressing a polygalacturonase-inhibiting protein from *Phaseolus vulgaris* (PvPGIP2) fused to a polygalacturonase from *Fusarium phyllophilum* (FpPG) under control of the XVE β-oestradiol-inducible promoter in Col-0 background | (Benedetti *et al.*, 2015) | PCR; qRT-PCR |
| *pPR1:OGM #2* |  | Transgenic line expressing a polygalacturonase-inhibiting protein from *Phaseolus vulgaris* (PvPGIP2) fused to a polygalacturonase from *Fusarium phyllophilum* (FpPG) under control of the *PATHOGENESIS-RELATED GENE 1* (*PR1*, *At2g14610*) promoter in Col-0 background | (Benedetti *et al.*, 2015) | PCR |
| *Mp10* (#7-5 and #9-5) |  | Independent transgenic lines expressing an N-Flag-tagged Mp10/CSP4 from *Myzus persicae* (MYZPE13164_O_EIv2.1_0182260) under control of a dexamethasone-inducible promoter in Col-0 background | This work | PCR; qRT-PCR |
| *eds1-2* x *Mp10* (#7-5 and #9-5) |  | Independent transgenic lines generated by cross of *eds1-2* and *Mp10* (#7-5 and #9-5) in Col-0 background | This work | PCR |

**Table S2** List of primer sequences used in this study

| **Gene** | **ID** | **Forward primer** | **Reverse primer** | **Application** |
| --- | --- | --- | --- | --- |
| *GAPDH* | *At1g13440* | TTGGTGACAACAGGTCAAGCA | AAACTTGTCGCTCAATGCAATC | qRT-PCR |
| *FRK1* | *At2g19190* | ATCTTCGCTTGGAGCTTCTC | TGCAGCGCAAGGACTAGAG | qRT-PCR |
| *CYP81F2* | *At5g57220* | TGGCTATGCGTAAACTCGTG | GGTAAACTTCAAAATGGTGGTCA | qRT-PCR |
| *PAD3* | *At3g26830* | TGCTCCCAAGACAGACAATG | GTTTTGGATCACGACCCATC | qRT-PCR |
| *PAD4* | *At3g52430* | TTGTCGATTCGAGACGAGTG | GATAAGCCGGGGAAAAGAAC | qRT-PCR |
| *BBE3*/*FOX1*/  *RET-OX* | *At1g26380* | AGGTTCTCGAACCCTAACAACA | GCACAGACGACACGTAAGAAAG | qRT-PCR |
| *UBQ5* | *At3g62250* | GTTAAGCTCGCTGTTCTTCAGT | TCAAGCTTCAACTCCTTCTTTC | qRT-PCR;  RT-PCR |
| *BAK1* | *At4g33430* | AGTTTTGCCAACACCAAGTTG | TTTGCCACTCTTCCCATCTC | RT-PCR; Sequencing |
| *BKK1* | *At2g13790* | AGAAAACCACAGGACCACTT | CTATATGGCCAATTGTACCG | RT-PCR |
| *CPK5* | *At4g35310* | TTTAGTTTCTCCTTTTAAGTCAG | TCGTTCCAAATTGACCTTGAC | RT-PCR |
| *CPK6* | *At2g17290* | GGCAATTCATGTCGTGGTT | AACAGAGAGACCAAAATCAATGG | RT-PCR |
| *CPK11* | *At1g35670* | GCCAAACCCTAGACGTCCTT | TAGTAGGCCACGGGTCAGAT | RT-PCR |
| *GRP3* | *At2g05520* | ATGGCTTCCAAGGCTTTGGT | TTAGTGACCGGGCTGAGTC | RT-PCR |
| *GRP3-RFP* | *At2g05520-RFP* fusion | GGAAGTGGGGGAAGTTACTG | GAGCCGTACTGGAACTGAGG | RT-PCR |
| *OGM* | *PvPGIP2*-*FpPG* fusion | CCTCCCAAAACCCTACCCTA | GAGAAGTCGAGGGTGACGAG | qRT-PCR;  PCR |
| *Mp10* | *MYZPE13164_O_EIv2.1_0182260* | GGTCGGAGCGCCGCAAAAAG | TTGGAACCCAAAACTTGGTCGATGT | qRT-PCR;  PCR |
| attB-*Flag*-*Mp10* | *Flag*-*Mp10* fusion | ggggacaagtttgtacaaaaaagcaggctATGGACTACAAAGACGATGACGACAagGCGCCGCAAAAAGAT | ggggaccactttgtacaagaaagctgggtTTAAAATTTGACAACACCTTTTTTC | Cloning |
| *EDS1* | *At3g48090* | TGTTTGTGAATTGACTGGAAGC | TCAGCCCAAAAGCATGATCC | PCR |

**Table S3** *Arabidopsis thaliana* genes and elements involved in both oligogalacturonide (OG) signalling and immunity to *Myzus persicae*

| **Gene/element name** | **References for OG signalling** | **References for immunity to *M. persicae*** |
| --- | --- | --- |
| *BAK1* | (Gravino *et al.*, 2017) | (Chaudhary *et al.*, 2014; Prince *et al.*, 2014; Vincent *et al.*, 2017; Tungadi *et al.*, 2021); This work |
| *BKK1* | (Gravino *et al.*, 2017) | (Tungadi *et al.*, 2021); This work |
| *CPK5* | (Gravino *et al.*, 2015) | This work |
| *CPK6* | (Gravino *et al.*, 2015) | This work |
| *CPK11* | (Gravino *et al.*, 2015) | This work |
| Ca^2+^ | (Moscatiello *et al.*, 2006) | (Vincent *et al.*, 2017) |
| *GRP3* | (Gramegna *et al.*, 2016) | This work |
| *EDS1* | This work | (Pegadaraju *et al.*, 2007; Liu *et al.*, 2025); This work |
| *RBOHD* | (Galletti *et al.*, 2008) | (Miller *et al.*, 2009) |
| *EIN2* | (Gravino *et al.*, 2015) | (Kettles *et al.*, 2013) |
| *PAD3* | (Ferrari *et al.*, 2007) | (Kettles *et al.*, 2013) |

**Table S4** Post hoc comparisons - genotype ✻ treatment, relative to Fig. 6

|  |  | **Mean Difference** | **SE** | **df** | **t** | **p_tukey_** | **Significance** |
| --- | --- | --- | --- | --- | --- | --- | --- |
| (Col-0 H₂O) | (Mp10 #7-5 H₂O) | 25.187 | 6.342 | 180 | 3.971 | 0.006 | ** |
|  | (Mp10 #9-5 H₂O) | 25.875 | 6.342 | 180 | 4.08 | 0.004 | ** |
|  | (eds1-2 H₂O) | 0.937 | 6.342 | 180 | 0.148 | 1 |  |
|  | (eds1-2 x Mp10 #7-5 H₂O) | -17.625 | 6.342 | 180 | -2.779 | 0.198 |  |
|  | (eds1-2 x Mp10 #9-5 H₂O) | -15.75 | 6.342 | 180 | -2.483 | 0.357 |  |
|  | (Col-0 OG) | 30.562 | 6.342 | 180 | 4.819 | < .001 | *** |
|  | (Mp10 #7-5 OG) | 25.438 | 6.342 | 180 | 4.011 | 0.005 | ** |
|  | (Mp10 #9-5 OG) | 27.125 | 6.342 | 180 | 4.277 | 0.002 | ** |
|  | (eds1-2 OG) | 0.437 | 6.342 | 180 | 0.069 | 1 |  |
|  | (eds1-2 x Mp10 #7-5 OG) | 12.25 | 6.342 | 180 | 1.931 | 0.738 |  |
|  | (eds1-2 x Mp10 #9-5 OG) | 11.25 | 6.342 | 180 | 1.774 | 0.83 |  |
| (Mp10 #7-5 H₂O) | (Mp10 #9-5 H₂O) | 0.687 | 6.342 | 180 | 0.108 | 1 |  |
|  | (eds1-2 H₂O) | -24.25 | 6.342 | 180 | -3.824 | 0.01 | ** |
|  | (eds1-2 x Mp10 #7-5 H₂O) | -42.813 | 6.342 | 180 | -6.75 | < .001 | *** |
|  | (eds1-2 x Mp10 #9-5 H₂O) | -40.938 | 6.342 | 180 | -6.455 | < .001 | *** |
|  | (Col-0 OG) | 5.375 | 6.342 | 180 | 0.847 | 0.999 |  |
|  | (Mp10 #7-5 OG) | 0.25 | 6.342 | 180 | 0.039 | 1 |  |
|  | (Mp10 #9-5 OG) | 1.937 | 6.342 | 180 | 0.305 | 1 |  |
|  | (eds1-2 OG) | -24.75 | 6.342 | 180 | -3.902 | 0.007 | ** |
|  | (eds1-2 x Mp10 #7-5 OG) | -12.938 | 6.342 | 180 | -2.04 | 0.666 |  |
|  | (eds1-2 x Mp10 #9-5 OG) | -13.938 | 6.342 | 180 | -2.198 | 0.554 |  |
| (Mp10 #9-5 H₂O) | (eds1-2 H₂O) | -24.938 | 6.342 | 180 | -3.932 | 0.007 | ** |
|  | (eds1-2 x Mp10 #7-5 H₂O) | -43.5 | 6.342 | 180 | -6.859 | < .001 | *** |
|  | (eds1-2 x Mp10 #9-5 H₂O) | -41.625 | 6.342 | 180 | -6.563 | < .001 | *** |
|  | (Col-0 OG) | 4.688 | 6.342 | 180 | 0.739 | 1 |  |
|  | (Mp10 #7-5 OG) | -0.437 | 6.342 | 180 | -0.069 | 1 |  |
|  | (Mp10 #9-5 OG) | 1.25 | 6.342 | 180 | 0.197 | 1 |  |
|  | (eds1-2 OG) | -25.438 | 6.342 | 180 | -4.011 | 0.005 | ** |
|  | (eds1-2 x Mp10 #7-5 OG) | -13.625 | 6.342 | 180 | -2.148 | 0.589 |  |
|  | (eds1-2 x Mp10 #9-5 OG) | -14.625 | 6.342 | 180 | -2.306 | 0.476 |  |
| (eds1-2 H₂O) | (eds1-2 x Mp10 #7-5 H₂O) | -18.562 | 6.342 | 180 | -2.927 | 0.141 |  |
|  | (eds1-2 x Mp10 #9-5 H₂O) | -16.687 | 6.342 | 180 | -2.631 | 0.271 |  |
|  | (Col-0 OG) | 29.625 | 6.342 | 180 | 4.671 | < .001 | *** |
|  | (Mp10 #7-5 OG) | 24.5 | 6.342 | 180 | 3.863 | 0.008 | ** |
|  | (Mp10 #9-5 OG) | 26.188 | 6.342 | 180 | 4.129 | 0.003 | ** |
|  | (eds1-2 OG) | -0.5 | 6.342 | 180 | -0.079 | 1 |  |
|  | (eds1-2 x Mp10 #7-5 OG) | 11.313 | 6.342 | 180 | 1.784 | 0.825 |  |
|  | (eds1-2 x Mp10 #9-5 OG) | 10.313 | 6.342 | 180 | 1.626 | 0.897 |  |
| (eds1-2 x Mp10 #7-5 H₂O) | (eds1-2 x Mp10 #9-5 H₂O) | 1.875 | 6.342 | 180 | 0.296 | 1 |  |
|  | (Col-0 OG) | 48.188 | 6.342 | 180 | 7.598 | < .001 | *** |
|  | (Mp10 #7-5 OG) | 43.063 | 6.342 | 180 | 6.79 | < .001 | *** |
|  | (Mp10 #9-5 OG) | 44.75 | 6.342 | 180 | 7.056 | < .001 | *** |
|  | (eds1-2 OG) | 18.062 | 6.342 | 180 | 2.848 | 0.169 |  |
|  | (eds1-2 x Mp10 #7-5 OG) | 29.875 | 6.342 | 180 | 4.71 | < .001 | *** |
|  | (eds1-2 x Mp10 #9-5 OG) | 28.875 | 6.342 | 180 | 4.553 | < .001 | *** |
| (eds1-2 x Mp10 #9-5 H₂O) | (Col-0 OG) | 46.313 | 6.342 | 180 | 7.302 | < .001 | *** |
|  | (Mp10 #7-5 OG) | 41.188 | 6.342 | 180 | 6.494 | < .001 | *** |
|  | (Mp10 #9-5 OG) | 42.875 | 6.342 | 180 | 6.76 | < .001 | *** |
|  | (eds1-2 OG) | 16.187 | 6.342 | 180 | 2.552 | 0.315 |  |
|  | (eds1-2 x Mp10 #7-5 OG) | 28 | 6.342 | 180 | 4.415 | 0.001 | ** |
|  | (eds1-2 x Mp10 #9-5 OG) | 27 | 6.342 | 180 | 4.257 | 0.002 | ** |
| (Col-0 OG) | (Mp10 #7-5 OG) | -5.125 | 6.342 | 180 | -0.808 | 1 |  |
|  | (Mp10 #9-5 OG) | -3.438 | 6.342 | 180 | -0.542 | 1 |  |
|  | (eds1-2 OG) | -30.125 | 6.342 | 180 | -4.75 | < .001 | *** |
|  | (eds1-2 x Mp10 #7-5 OG) | -18.313 | 6.342 | 180 | -2.887 | 0.154 |  |
|  | (eds1-2 x Mp10 #9-5 OG) | -19.313 | 6.342 | 180 | -3.045 | 0.104 |  |
| (Mp10 #7-5 OG) | (Mp10 #9-5 OG) | 1.687 | 6.342 | 180 | 0.266 | 1 |  |
|  | (eds1-2 OG) | -25 | 6.342 | 180 | -3.942 | 0.006 | ** |
|  | (eds1-2 x Mp10 #7-5 OG) | -13.188 | 6.342 | 180 | -2.079 | 0.638 |  |
|  | (eds1-2 x Mp10 #9-5 OG) | -14.188 | 6.342 | 180 | -2.237 | 0.525 |  |
| (Mp10 #9-5 OG) | (eds1-2 OG) | -26.688 | 6.342 | 180 | -4.208 | 0.002 | ** |
|  | (eds1-2 x Mp10 #7-5 OG) | -14.875 | 6.342 | 180 | -2.345 | 0.449 |  |
|  | (eds1-2 x Mp10 #9-5 OG) | -15.875 | 6.342 | 180 | -2.503 | 0.345 |  |
| (eds1-2 OG) | (eds1-2 x Mp10 #7-5 OG) | 11.813 | 6.342 | 180 | 1.862 | 0.78 |  |
|  | (eds1-2 x Mp10 #9-5 OG) | 10.813 | 6.342 | 180 | 1.705 | 0.864 |  |
| (eds1-2 x Mp10 #7-5 OG) | (eds1-2 x Mp10 #9-5 OG) | -1 | 6.342 | 180 | -0.158 | 1 |  |

** p < .01, *** p < .001

*Note.* P-value adjusted for comparing a family of 12 estimates.

**Table S5** List of icons, including attribution and license, used to generate the model in Fig. 8

| **Icon** | **Attribution** | **License** |
| --- | --- | --- |
| aphid | DBCLS https://togotv.dbcls.jp/en/pics.html | CC-BY 4.0 Unported https://creativecommons.org/licenses/by/4.0/ |
| cell_group | JhonnyXC https://github.com/JRider16 | CC0 https://creativecommons.org/publicdomain/zero/1.0/ |
| channel-membrane-red-2 | Servier https://smart.servier.com/ | CC-BY 3.0 Unported https://creativecommons.org/licenses/by/3.0/ |
| Lightning bolt | <a href="https://www.vecteezy.com/free-vector/lightning-bolt">Lightning Bolt Vectors by Vecteezy</a> | N/A |

N/A, not available

*Note.* All the material in 4.0 license version was modified.

**Method S1** Quantification of DAB staining in *Arabidopsis thaliana* leaves using the Fiji plugin Colour Deconvolution 2

1. **Install the plugin**
   Download the Colour Deconvolution2 plugin from [this GitHub repository](https://github.com/landinig/IJ-Colour_Deconvolution2/blob/main/colour_deconvolution2.jar) and install it in Fiji.
2. **Open the image**
   Restart Fiji and open an image of interest containing DAB-stained leaves and a ruler.
3. **Run the plugin**
   Go to *Image* → *Color* → *Colour Deconvolution2*.
4. **Configure plugin settings**
   In the Colour Deconvolution2 window:
   - Select **Vector**: *H DAB* (haematoxylin and DAB)
   - Select Output: *8bit_Transmittance*
   - Check the following options:
     - ***Simulated LUTs***
     - ***Cross product for Colour 3***
     - ***Show matrices***
     - ***Hide legend***
   - Click **OK**
5. **Select the appropriate image**
   After colour deconvolution, the image is subdivided into three colours:
   - **Colour 1**: Haematoxylin
   - **Colour 2**: DAB (this is the one used for quantification)
   - **Colour 3**: a third vector orthogonal to the first two (this is not a real colour but the residual of the deconvolution process)

Use **Colour 2** for DAB quantification.

1. **Apply thresholding** (see Notes 1, 2)
   Go to *Image* → *Adjust* → *Threshold*. In the Threshold window:
   - Select ***Default*** method
   - Select ***Red*** colour
   - Check the ***Dark background* option**
   - Set the **left slider** to 0
   - Adjust the **right slider** until the DAB signal appears red and the background is white
2. **Set the scale**
   - Draw a straight line over the ruler
   - Go to *Analyse* → *Set Scale*
   - Enter the known distance and unit (e.g., 1 cm) in the *Set Scale* window, then click **OK**
3. **Set measurements**
   Go to *Analyse* → *Set Measurements*. In the Set Measurements window:
   - Check ***Area*** and ***Area Fraction***
   - Set the number of ***Decimal Places* to 9**
   - Click **OK**
4. **Define region of interest (ROI)**
   Use the Polygon Selections tool to draw an ROI around an entire leaf, excluding the petiole.
5. **Measure the ROI**
   Go to *Analyse* → *Measure*.
6. **Export results**
   Save the measurement results or copy and paste them into Excel for further analysis.

### **Notes**

1. To ensure consistent thresholding, all replicate leaves belonging to different genotypes to be compared within an experiment should be captured in the **same image**.
2. Maintain consistent lighting conditions across independent experiments to ensure reliable comparisons.

**References**

**Bartsch M, Gobbato E, Bednarek P, Debey S, Schultze JL, Bautor J, Parker JE. 2006.** Salicylic acid-independent ENHANCED DISEASE SUSCEPTIBILITY1 signaling in Arabidopsis immunity and cell death is regulated by the monooxygenase FMO1 and the Nudix hydrolase NUDT7. *Plant Cell* **18**(4): 1038-1051.

**Benedetti M, Mattei B, Pontiggia D, Salvi G, Savatin DV, Ferrari S. 2017.** Methods of isolation and characterization of oligogalacturonide elicitors. In: Shan L, He P, (eds) *Plant pattern recognition receptors*. *Methods Molecular Biology* **1578**: 25-38.

**Benedetti M, Pontiggia D, Raggi S, Cheng Z, Scaloni F, Ferrari S, Ausubel FM, Cervone F, De Lorenzo G. 2015.** Plant immunity triggered by engineered in vivo release of oligogalacturonides, damage-associated molecular patterns. *Proc. Natl. Acad. Sci. U.S.A.* **112**(17): 5533-5538.

**Benedetti M, Verrascina I, Pontiggia D, Locci F, Mattei B, De Lorenzo G, Cervone F. 2018.** Four Arabidopsis berberine bridge enzyme-like proteins are specific oxidases that inactivate the elicitor-active oligogalacturonides. *Plant Journal* **94**(2): 260-273.

**Boudsocq M, Willmann MR, McCormack M, Lee H, Shan L, He P, Bush J, Cheng SH, Sheen J. 2010.** Differential innate immune signalling via Ca^2+^ sensor protein kinases. *Nature* **464**(7287): 418-422.

**Chaudhary R, Atamian HS, Shen Z, Briggs SP, Kaloshian I. 2014.** GroEL from the endosymbiont Buchnera aphidicola betrays the aphid by triggering plant defense. *Proc Natl Acad Sci USA* **111**(24): 8919-8924.

**Ferrari S, Galletti R, Denoux C, De Lorenzo G, Ausubel FM, Dewdney J. 2007.** Resistance to *Botrytis cinerea* induced in Arabidopsis by elicitors is independent of salicylic acid, ethylene, or jasmonate signaling but requires PHYTOALEXIN DEFICIENT3. *Plant Physiol.* **144**(1): 367-379.

**Galletti R, Denoux C, Gambetta S, Dewdney J, Ausubel FM, De Lorenzo G, Ferrari S. 2008.** The AtrbohD-mediated oxidative burst elicited by oligogalacturonides in Arabidopsis is dispensable for the activation of defense responses effective against *Botrytis cinerea*. *Plant Physiol.* **148**(3): 1695-1706.

**Gramegna G, Modesti V, Savatin DV, Sicilia F, Cervone F, De Lorenzo G. 2016.** GRP-3 and KAPP, encoding interactors of WAK1, negatively affect defense responses induced by oligogalacturonides and local response to wounding. *Journal of Experimental Botany* **67**(6): 1715-1729.

**Gravino M, Locci F, Tundo S, Cervone F, Savatin DV, De Lorenzo G. 2017.** Immune responses induced by oligogalacturonides are differentially affected by AvrPto and loss of BAK1/BKK1 and PEPR1/PEPR2. *Molecular Plant Pathology* **18**(4): 582-595.

**Gravino M, Savatin DV, Macone A, De Lorenzo G. 2015.** Ethylene production in *Botrytis cinerea*- and oligogalacturonide-induced immunity requires calcium-dependent protein kinases. *Plant Journal* **84**(6): 1073-1086.

**Kettles GJ, Drurey C, Schoonbeek HJ, Maule AJ, Hogenhout SA. 2013.** Resistance of *Arabidopsis thaliana* to the green peach aphid, *Myzus persicae*, involves camalexin and is regulated by microRNAs. *New Phytol* **198**(4): 1178-1190.

**Kumar S, Stecher G, Li M, Knyaz C, Tamura K. 2018.** MEGA X: Molecular Evolutionary Genetics Analysis across Computing Platforms. *Mol Biol Evol* **35**(6): 1547-1549.

**Liu Q, Neefjes A, Singh A, Kobylinska R, Mugford S, Marzo M, Canham J, Schuster M, van der Hoorn R, Chen Y, et al. 2025.** Aphid effectors suppress plant immunity via recruiting defense proteins to processing bodies. *Sci. Adv.* **11**(29):eadv1447.

**Miller G, Schlauch K, Tam R, Cortes D, Torres MA, Shulaev V, Dangl JL, Mittler R. 2009.** The plant NADPH oxidase RBOHD mediates rapid systemic signaling in response to diverse stimuli. *Sci.Signal.* **2**(84): ra45.

**Moscatiello R, Mariani P, Sanders D, Maathuis FJM. 2006.** Transcriptional analysis of calcium-dependent and calcium-independent signalling pathways induced by oligogalacturonides. *J. Exp. Bot.* **57**(11): 2847-2865.

**Pegadaraju V, Louis J, Singh V, Reese JC, Bautor J, Feys BJ, Cook G, Parker JE, Shah J. 2007.** Phloem-based resistance to green peach aphid is controlled by Arabidopsis PHYTOALEXIN DEFICIENT4 without its signaling partner ENHANCED DISEASE SUSCEPTIBILITY1. *Plant J* **52**(2): 332-341.

**Prince DC, Drurey C, Zipfel C, Hogenhout SA. 2014.** The leucine-rich repeat receptor-like kinase BRASSINOSTEROID INSENSITIVE1-ASSOCIATED KINASE1 and the cytochrome P450 PHYTOALEXIN DEFICIENT3 contribute to innate immunity to aphids in Arabidopsis. *Plant Physiol.* **164**(4): 2207-2219.

**Schwessinger B, Roux M, Kadota Y, Ntoukakis V, Sklenar J, Jones A, Zipfel C. 2011.** Phosphorylation-dependent differential regulation of plant growth, cell death, and innate immunity by the regulatory receptor-like kinase BAK1. *PLoS Genetics* **7**(4): e1002046.

**Tungadi T, Watt LG, Groen SC, Murphy AM, Du Z, Pate AE, Westwood JH, Fennell TG, Powell G, Carr JP. 2021.** Infection of Arabidopsis by cucumber mosaic virus triggers jasmonate-dependent resistance to aphids that relies partly on the pattern-triggered immunity factor BAK1. *Mol Plant Pathol* **22**(9): 1082-1091.

**Vincent TR, Avramova M, Canham J, Higgins P, Bilkey N, Mugford ST, Pitino M, Toyota M, Gilroy S, Miller AJ, et al. 2017.** Interplay of plasma membrane and vacuolar ion channels, together with BAK1, elicits rapid cytosolic calcium elevations in Arabidopsis during aphid feeding. *Plant Cell* **29**(6): 1460-1479.
